# HEV ORF3 downregulates TLR7 to inhibit the generation of type I interferon via impairment of multiple signaling pathways

**DOI:** 10.1038/s41598-018-26975-4

**Published:** 2018-06-05

**Authors:** Qingsong Lei, Lin Li, Shujun Zhang, Tianju Li, Xiaomei Zhang, Xiaolin Ding, Bo Qin

**Affiliations:** 1 Chongqing Key Laboratory of Infectious Diseases and Parasitic Diseases,Department of Infectious Diseases, the First Affiliated Hospital of Chongqing Medical University, Chongqing, 400016 China; 2 Department of hepatic diseases, Chongqing Tranditional Chinese Medicine Hospital, Chongqing, 400011 China

## Abstract

Hepatitis E is the most common type of acute hepatitis prevalent worldwide. The open reading frame 3 protein of HEV (HEV ORF3) is proposed to create a favorable environment for viral replication and pathogenesis. However, the mechanisms by which HEV overcomes the effects of host immunity, particularly the role of ORF3, remain to be established. Expression of IFNα and IFNβ in supernatant and cell samples was examined via ELISA and quantitative RT-PCR. The protein levels of specific signaling factors in cells overexpressing HEV ORF3 were examined via western blot. Analyses of cells transfected with vectors expressing ORF3 demonstrated that HEV ORF3 significantly impairs the generation of endogenous type I interferon through downregulating TLR3 and TLR7 as well as their corresponding downstream signaling pathways. Moreover, inhibition of NFκB, JAK/STAT and JNK/MAPK signaling pathways contributed significantly to suppression of increased levels of TLR7. Levels of p-P65, p-STAT1 and p-JNK were markedly impaired in ORF3-expressing cells, even upon treatment with the respective agonists. HEV ORF3 inhibits the production of endogenous type I interferon through downregulation of TLR3 and TLR7. Furthermore, suppression of TLR7 is achieved through impairment of multiple signaling pathways, including NFκB, JAK/STAT and JNK/MAPK.

## Introduction

Hepatitis E virus (HEV) is the most common causative agent of acute hepatitis worldwide, often leading to chronic hepatitis or fulminant hepatic failure in immunocompromised individuals and pregnant women^1,2^. Global hepatitis report 2017of WHO showed that there are an estimated 20 million HEV infections worldwide every year, leading to an estimated 3.3 million symptomatic cases of acute hepatitis E. HEV is a plus-stranded RNA virus composed of three open reading frames^3,4^. ORF1 encodes a nonstructural polyprotein involved in replication progression of HEV, ORF2 encodes the capsid protein responsible for virion assembly and immunogenicity of virus and ORF3 encodes a small multifunctional phosphoprotein^5^. All three ORFs of HEV regulate numerous cellular signaling pathways and inhibit host immune responses to promote survival of infected cells^6^. A number of studies suggest a key role of HEV ORF3 in manipulating various host cell processes during viral infection and propagation. Interactions of ORF3 with host proteins are proposed to create a favorable environment for HEV replication and pathogenesis^7,8^.

The mechanism by which HEV overcomes the effects of cellular immunity in host cells, in particular, the role of ORF3, are yet to be established. ORF3 is proposed to play critical roles in immune evasion by HEV. Earlier, Xu and co-workers showed that ORF3 transiently activates nuclear factor kappa B (NFκB) signaling at the early infection and conversely inhibits this pathway at the late phase to create a favorable replication environment for HEV^9^. HEV inhibits IFNα signaling through binding of the ORF3 protein to signal transducer and activator of transcription (STAT1) in the human alveolar epithelial cell line A54910. Additionally, the P2 domain of ORF3 of ORF3 can downregulate TLR3-mediated NFκB signaling via inhibition of TRADD and RIP1 by binding with Lys377, the functional ubiquitination site of RIP111.

The innate immune system is the major contributor to acute inflammation induced by microbial infection. In addition to the important roles of macrophages and dendritic cells (DCs), non-professional cells are crucial for activation of the innate immune system considering their amount and the expression of Pattern recognition receptors^10^. Pattern recognition receptors, especially the Toll-like receptor (TLR) family, are key players in the initiation of immune cell activity and innate immune responses^11^. The type I interferon (IFN) system, which includes IFNα and IFNβ mainly induced by TLRs, is an innate immune response. Upon recognition of respective PAMP, TLRs recruit TIR adaptors that initiate downstream signaling events containing TRAF and IRAK that lead to the secretion of type I interferon and inflammatory cytokines^12^.

Viral infection thus initiates the innate antiviral immune response through TLRs^13^. Cells readily secrete IFNα/β as part of the biological defense mechanisms that play a primary role in viral restriction. In turn, IFNα/β triggers the synthesis of a range of antiviral proteins, which serve as cell-autonomous intrinsic restriction factors^14^. The interferon (IFN)-induced anti-viral response is among the earliest and most potent of the innate immune responses. However, viruses have evolved multiple strategies to evade the type I IFN response, which promote their escape from host immunity and spread of infection^15^. For instance, HEV replication in hepatoma cells is reported to inhibit poly I:C-induced IFNβ expression^16^. Additionally, HEV not only downregulates RIG-I helicase-like receptor-mediated IFN induction but also employs MAVS in curtailing the host inflammatory response^10^.

While inspiring advances have been made in the prevention and treatment of hepatitis E^17,18^, the mechanism underlying inhibition of the host immune response by HEV ORF3 remains to be established. A previous study by our group showed that ORF3 inhibits secretion of inflammatory factors of THP1 macrophages by suppressing activation of the NFκB pathway^19^. The current investigation focused on the effects of HEV ORF3 on endogenous type I interferon generation and associated mechanisms. Our data provide a new perspective on cellular responses to HEV infection and insights into the molecular mechanisms of HEV pathogenesis and innate immunity.

## Materials and Methods

### Cell Culture

THP1 monocytes (ATCC, Manassas, VA, USA) and HepG2 cells (Cell Bank of the Chinese Academy of Sciences, Shanghai, China), authenticated by STR profiling (Supplement S1,S2), were cultured in RPMI 1640 supplemented with 10% fetal bovine serum (FBS, Gibco). Huh7 and normal hepatocytes LO2 cell lines (stored at Chongqing Key Laboratory of Infectious Diseases and Parasitic Diseases) were cultured in Dulbecco’s Modified Eagle Medium (DMEM, Gibco) containing 10% FBS. Adenovirus vector (Ad-Hu5) and plasmid vectors (pcDNA3.1-GFP) overexpressing HEV ORF3 gene (genotype 1 strain Sar55) were constructed in this study. THP1 macrophages differentiated from monocytes by using 10 ng/mL phorbol12-myristate13-acetate (PMA; Sigma) were transduced with recombinant adenovirus vector expressing ORF3 protein (Ad-ORF3) or control vector (Ad-Hu5) at a MOI of 20. Plasmids premixed with Lipofectamine 2000 (Sigma) were transfected into LO2 cells. Lipopolysaccharide (LPS; Sigma) (100 ng/mL) was added to cells to induce expression of TLR7. Cells were divided into different groups based on treatments with agonists and inhibitors.

### Enzyme-linked Immunosorbent Assay (ELISA)

All collected supernatant samples were stored at −40 °C and assessed using IFNα and IFNβ ELISA kits according to the manufacturer’s protocols (NeoBioscience Technology Co., Ltd., Shenzhen, China).

### RNA Extraction and Quantitative RT-PCR

Total RNA was isolated from different groups using TRIzol reagent (Invitrogen) and further purified with the RNeasy Mini kit (Qiagen) according to the manufacturer’s instructions. RNA samples were assessed with the NanoDrop 2000 spectrophotometer (Thermo Fisher Scientific, MA, USA) and Agilent 2100 Bioanalyzer (Agilent Technologies, CA, USA). Complementary DNA (cDNA) was synthesized using the PrimeScript^TM^ RT Reagent Kit (TaKaRa, DaLian, China). Quantitative real-time PCR (qRT-PCR) was performed using the Light Cycler system (Bio-Rad Laboratories, Inc., Hercules, CA). The reaction mixture contained 5 μL SYBR Green Master Mix (SYBR Premix Ex Taq^TM^ II Kit, TaKaRa), 1 μL cDNA template, different reverse and forward primers at a final concentration of 0.5 μM and RNase-free H_2_O (Table 1). The PCR conditions were as follows: 10 min at 95 °C followed by 40 cycles of 95 °C for 15 s and 60 °C for 30 s. Gene expression was analyzed via comparative 2−ΔΔCt and normalized to that of beta-actin.

**Table 1:** Primers for quantitative real-time PCR.

Gene		Sequence 5′–3′
β-actin	Reverse	GCAAGCAGGAACGATGAG
	Forward	CCATGCCAATGTTGTCTCTT
IFNα	Reverse	GTGAGGAAATACTTCCAAAGAATCAC
	Forward	TCTCATGATTTCTGCTCTGACAA
IFNβ	Reverse	GCCGCATTGACCATGTATGAGA
	Forward	GAGATCTTCAGTTTCGGAGGTAAC
TLR3	Reverse	TTTGCAAGAGGAATGTTTAAATCT
	Forward	CACCTATCCGTTCTTTCTGAACTG
TLR4	Reverse	TCTTGGTGGAAGTTGAACG
	Forward	GCCACACCGGGAATAA
TLR7	Reverse	GGAAATTGCCCTCGTTGTTA
	Forward	CTTTTCACCCAGGCAGAATC

### Western Blot

Protein lysates extracted from different groups were separated via 8–10% sodium dodecyl sulfate polyacrylamide gel electrophoresis (SDS-PAGE) in Tris/glycine buffer (25 mM Tris and 250 mM glycine). The primary antibodies specific for TLR3 (115–130 kD), TLR4 (100–135 kD), TLR7 (140 kD), p-STAT1 (84, 91 kD), STAT1 (84, 91 kD), p-ERK1/2 (42, 44 kD), ERK1/2 (42, 44 kD), p-P38 (43 kD), P38 (43 kD), p-JNK1/2 (46, 54 kD), JNK1/2 (46, 54 kD), p-P65 (65 kD) and P65 (65 kD) were purchased from Cell Signaling Technology. HEV ORF3 (13.5 kD), p-IRF3 (47 kD), IRF3 (47 kD), p-IRF7 (54 kD) and IRF7 (54 kD) antibodies were acquired from Bioss (Beijing, China) and GFP (27 kD) from Wanleibio (Jilin, China). GAPDH (36 kD) antibody was purchased from Proteintech Group (Wuhan, China). Secondary antibodies were obtained from Pierce (1:5000 dilution). Bands were transferred to polyvinylidene fluoride (PVDF, Millipore, Billerica, MA) and proteins detected using the enhanced ECL chemiluminescence detection kit (Millipore, Billerica, MA) on a ChemiDoc XRS + imaging system (Bio-Rad, CA, USA).

### Statistical analysis

Each experiment was repeated at least three times and data presented as means ± standard deviation (SD). Statistical analyses were performed using SPSS 19.0 software (SPSS Inc., Chicago, IL, USA) and Student’s *t*-test applied to compare the significance of differences between groups. *P* values ≤ 0.05 were considered statistically significant. Graphs were generated using GraphPad software (version 5.01, San Diego, CA, USA).

## Results

### HEV ORF3 impairs the generation of endogenous type I interferon in host cells through downregulating TLR3 and TLR7

**Figure 1:**
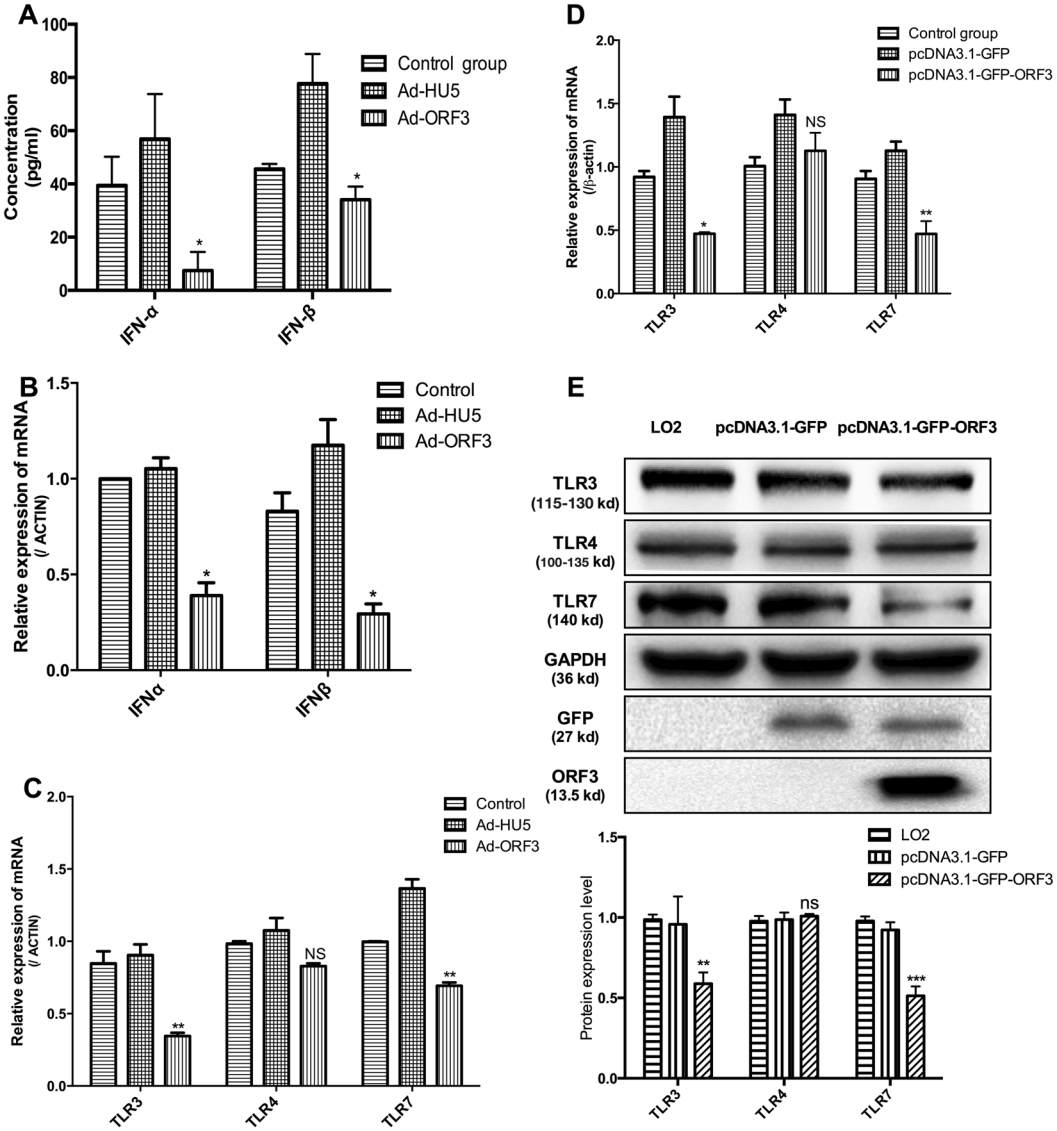
HEV ORF3 impairs endogenous type I interferon production in host cells. Adenovirus and plasmid vectors expressing ORF3 protein were transfected into THP1 and LO2 cell lines, respectively. (**A**) ELISA data showing that IFNα and IFNβ levels in THP1 cells expressing ORF3 protein are significantly decreased (P_α_ = 0.0133, P_β_ = 0.042). (**B**) mRNA levels of type I interferon were inhibited in ORF3-expressing cells (P_α_ = 0.0272, P_β_ = 0.0251). (**C**) The TLR4 level was not changed while TLR3 and TLR7 levels were significantly reduced in THP1 cells infected with Ad-ORF3 (P_TLR3_ = 0.0087, P_TLR4_ = 0.1185, P_TLR7_ = 0.0038). (**D**) Expression of TLR3, TLR4 and TLR7 was confirmed in LO2 cells transfected with pcDNA3.1-GFP-ORF3 (P_TLR3_ = 0.0285, P_TLR4_ = 0.3297, P_TLR7_ = 0.0054). (**E**) Protein levels of TLR3, TLR4 and TLR7 in LO2 cells were detected via western blot. Lane 1: Normal control LO2 cells. Lane 2: LO2 cells transfected with pcDNA3.1-GFP. Lane 3: LO2 cells transfected with pcDNA3.1-GFP-ORF3. Results are presented as means ± SD. Error bars indicate SD. *P < 0.05, **P < 0.01. All of full-length gels and blots are included in Supplement S3.

To ascertain whether HEV ORF3 can regulate innate immunity and generation of endogenous interferon of host cells, adenoviruses and plasmid vectors expressing ORF3 protein were transfected into THP1 and LO2 cell lines, respectively, and expression levels of type I interferon detected. Data from ELISA experiments showed a significant decrease in both IFNα and IFNβ protein levels in THP1 cells expressing ORF3 protein (Fig. 1A). Consistently, the mRNA levels of type I interferon were markedly decreased, supporting the theory that HEV ORF3 inhibits production of type I interferon (Fig. 1B). TLRs present the main intrinsic immune signaling pathway to generate IFN. Specifically, TLR3, TLR4 and TLR7 promote generation of IFN by identifying corresponding substances to activate homologous downstream signal molecules. Moreover, TLR4 expression was not significantly altered while TLR3 and TLR7 were markedly reduced in ORF3-expressing cells at both the mRNA and protein levels (Fig. 1C–E).

**Figure 2:**
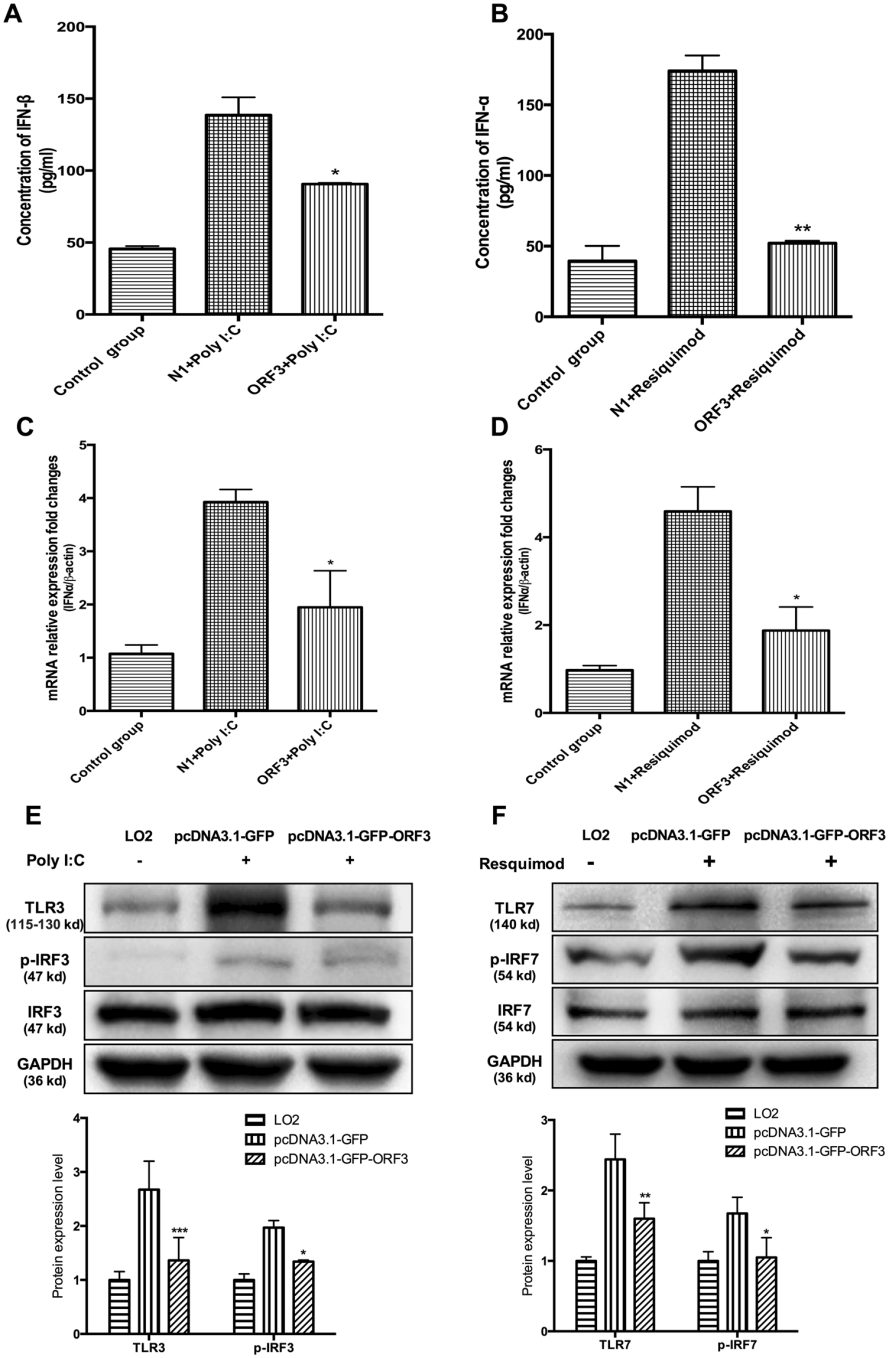
HEV ORF3 suppresses type I interferon through downregulating TLR3 and TLR7. Poly I:C and resiquimod, specific agonists for TLR3 and TLR7, were added into cells at concentrations of 10 µg/ml and 50 nM, respectively. (**A**) IFNβ protein expression was significantly lower in ORF3-expressing cells pretreated with poly I:C (*P* = 0.0185). (**B**) IFNα expression was significantly lower in ORF3-expressing cells pretreated with resiquimod (*P* = 0.0019). (**C**) IFNβ mRNA expression was significantly decreased in ORF3-expressing cells pretreated with poly I:C (*P* = 0.0245). (**D**) IFNα mRNA expression was significantly decreased in ORF3-expressing cells pretreated with resiquimod (P = 0.0499). (**E**) Expression of TLR3 and activation of p-IRF3 were markedly inhibited in ORF3-expressing cells pretreated with poly I:C. (**F**) Expression of TLR7 and activation of p-IRF7 were markedly inhibited in ORF3-expressing cells pretreated with resiquimod. Lane 1: Normal LO2 cells. Lane 2: LO2 cells transfected with pcDNA3.1-GFP pretreated with poly I:C or resiquimod. Lane 3: LO2 cells transfected with pcDNA3.1-GFP-ORF3 pretreated with poly I:C or resiquimod. All of full-length gels and blots are included in Supplement S3.

Poly I:C and resiquimod (specific agonists for TLR3 and TLR7, respectively) were added into LO2 cells, including those overexpressing ORF3, to determine the specific functions of HEV ORF3 protein. As shown in Fig. 2, the ORF3 expression impaired the induction of IFNα and IFNβ in LO2 cells pretreated with resiquimod or Poly I:C (Fig. 2A–D). Simultaneously, the relevant signaling molecules in TLR3 and TLR7 pathways were detected via western blot. Expressions of TLR3 and TLR7 proteins as well as active phosphorylated form of IRF3 and IRF7 were significantly inhibited in ORF3-expressing cells (Fig. 2E,F). Our results collectively suggest that HEV ORF3 impairs endogenous type I interferon production in host cells through downregulating TLR3 and TLR7 as well as their downstream signaling components tested.

### Multiple signaling pathways, including NFκB, JAK/STAT and JNK/MAPK, are crucial for expression of TLR7

**Figure 3:**
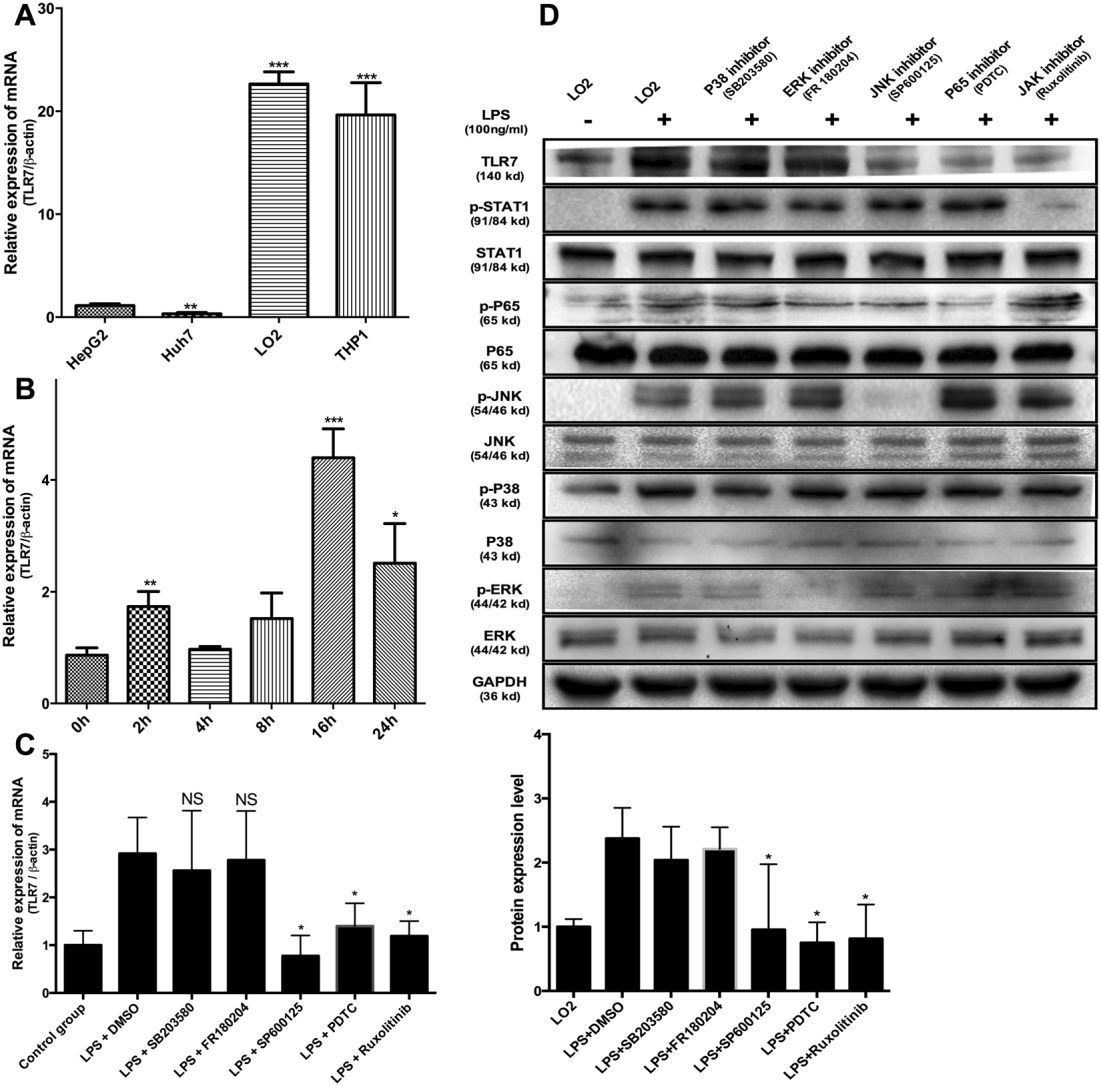
Multiple signaling pathways, including NFκB, JAK/STAT and JNK/MAPK, are crucial for expression of TLR7. (**A**) TLR7 mRNA expression in different cell lines. (**B**) TLR7 mRNA levels at different time-points after treatment with LPS. (**C**) LO2 cells pretreated with inhibitors specific for different pathways were stimulated with LPS for a further 18 h. Inhibition of NFκB, JAK/STAT and JNK/MAPK (*P* = 0.0423, 0.0216, 0.0129, respectively) signaling pathways led to significant suppression of TLR7 expression. On the other hand, regulation of ERK/MAPK and P38/MAPK (*P* = 0.6952, 0.8642) pathways had no significant inhibitory effect on TLR7 expression induced by LPS. (**D**) The expression of TLR7 in different groups verified that inhibition of NFκB, JAK/STAT and JNK/MAPK signaling pathways could significantly inhibit the increase in TLR7 expression, and ERK/MAPK and P38/MAPK pathways had no significant effect on the increased expression of TLR7 induced by LPS. Lane 1: Normal LO2 cells. Lanes 2–7: LO2 cells treated with LPS. Lane 3: Pretreatment with 5 µM SB203580. Lane 4: Pretreatment with 5 µM FR180204. Lane 5: Pretreatment with 5 µM SP600125. Lane 6: Pretreatment with PDTC. Lane 7: Pretreatment with 1 µM Ruxolitinib. All of full-length gels and blots are included in Supplement S3.

ORF3 protein is reported to induce downregulation of TLR3-mediated NFκB signaling via TRADD and RIP1^20^. Here, we focused on ORF3-mediated regulation of TLR7 expression. While the regulatory mechanisms of TLR7 expression are unknown, LPS is reported to induce TLR7 expression and generation of endogenous interferon^21^. Multiple signaling pathways (including NFκB, Janus kinase/signal transducer and activator of transcription (JAK/STAT) and mitogen-activated protein kinase (MAPK)), known to be activated by LPS combined with CD14 are recognized by TLR4. The LO2 cell line was used as a model for subsequent studies, in view of its abundant expression of TLR7 (Fig. 3A) and further increase in TLR7 mRNA levels upon treatment with LPS (Fig. 3B). To further clarify the upstream signaling pathways of TLR7, cells pretreated with specific inhibitors for different pathways were stimulated with LPS. Notably, inhibition of NFκB, JAK/STAT and c-Jun N-terminal kinase (JNK) signaling pathways led to a significant decrease in TLR7 expression, while suppression of ERK/MAPK and P38/MAPK pathways had no marked effects on TLR7 induced by LPS (Fig. 3C,D).

**Figure 4:**
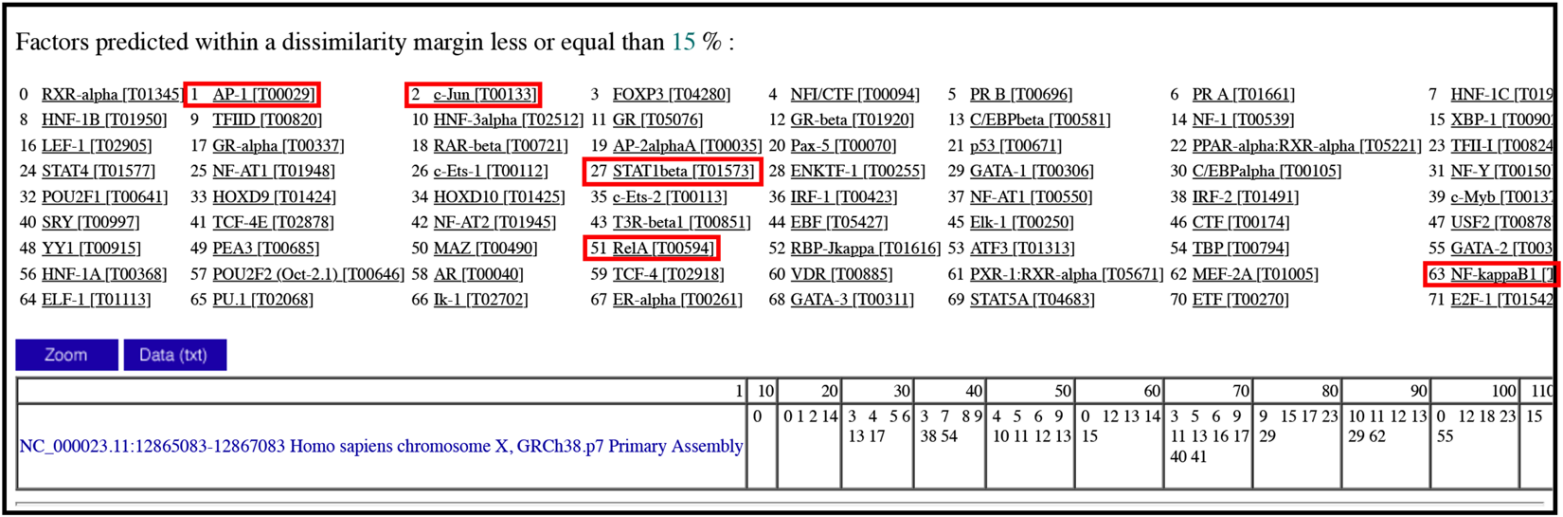
Potential transcription factors binding to the *TLR7* promoter region. Prediction of binding sites and potential transcription factors of the TLR7 promoter region (NC_000023.11:12865083–12867083) was performed using PROMO (http://alggen.lsi.upc.es/cgi-bin/promo). AP-1, c-Jun, STAT1beta, RelA, and NF-kappaB1 were identified as potential transcription factors that activate the *TLR7* gene promoter.

The binding sites and potential transcription factors of the TLR7 promoter region (NC_000023.11:12865083–12867083) were further predicted using PROMO (http://alggen.lsi.upc.es/cgi-bin/promo). The transcription factors AP-1, c-Jun, STAT1beta, RelA and NF-kappaB1 were identified as potential activators of the TLR7 promoter (Fig. 4). These findings indicate that multiple signaling pathways, including NFκB, JAK/STAT and JNK/MAPK, contribute to regulation of TLR7 expression.

### HEV ORF3 impairs activation of NFκB, JAK/STAT and JNK/MAPK pathways

**Figure 5:**
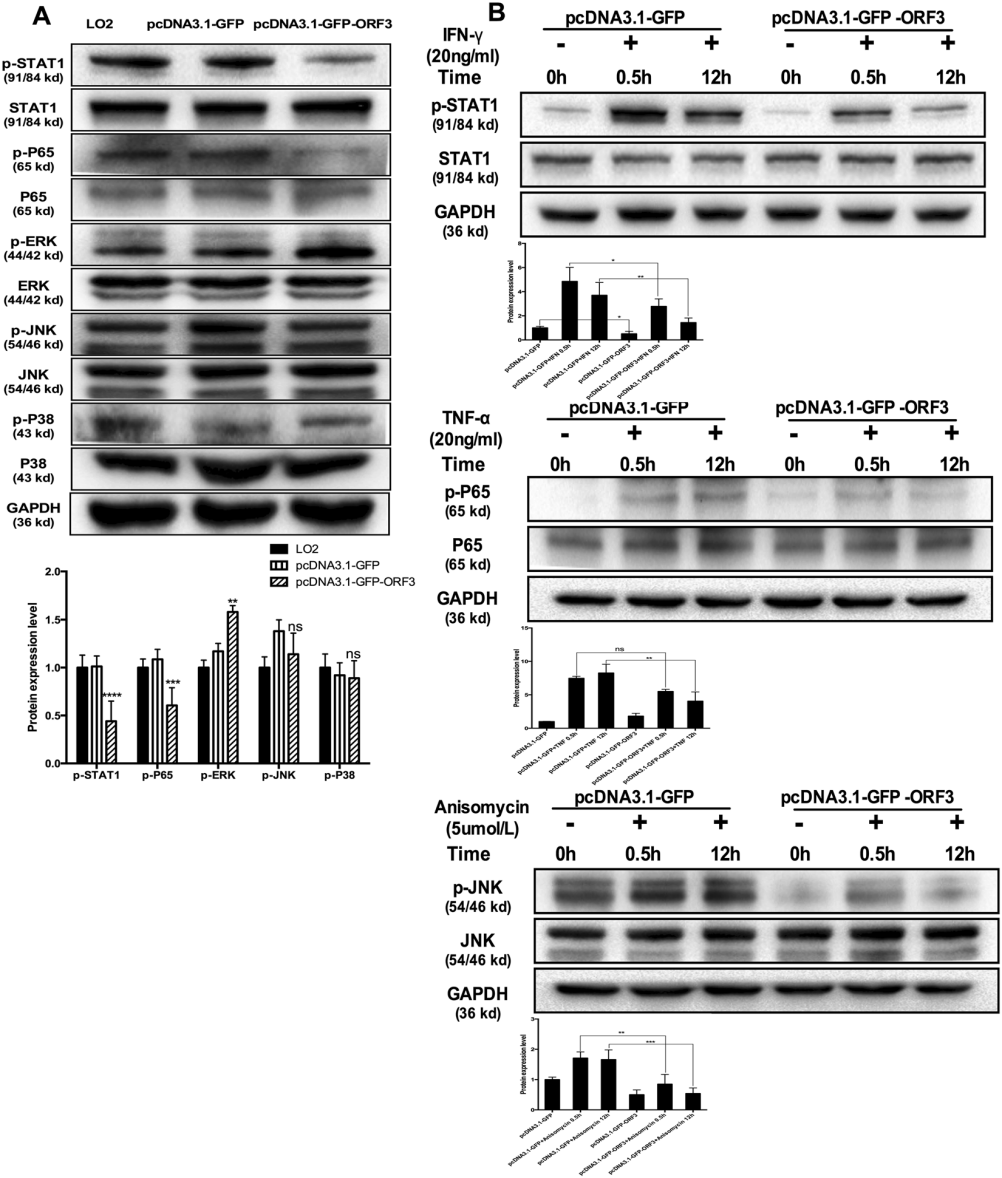
HEV ORF3 impairs the activation of NFκB, JAK/STAT and JNK/MAPK pathways. (**A**) HEV ORF3 strengthen p-ERK and inhibits p-P65, p-STAT1 and p-JNK, but does not affect p-P38. Lane 1: LO2 cells. Lane 2: LO2 cells transfected with pcDNA3.1-GFP. Lane 3: LO2 cells transfected with pcDNA3.1-GFP-ORF3. (**B**) Activation of p-P65, p-STAT1 and p-JNK was significantly impaired in ORF3-expressing cells treated with different agonists. Lane 1: LO2 cells transfected with pcDNA3.1-GFP. Lane 2: LO2 cells transfected with pcDNA3.1-GFP treated with different agonists for 0.5 h. Lane 3: LO2 cells transfected with pcDNA3.1-GFP treated with different agonists for 12 h. Lane 4: LO2 cells transfected with pcDNA3.1-GFP-ORF3. Lane 2: LO2 cells transfected with pcDNA3.1-GFP-ORF3 treated with different agonists for 0.5 h. Lane 3: LO2 cells transfected with pcDNA3.1-GFP-ORF3 treated with different agonists for 12 h. All of full-length gels and blots are included in Supplement S3.

ORF3 is a phosphorylated protein that regulates multiple signaling pathways in host cells. Our results showed that ORF3 strengthens p-ERK and inhibits activation of p-P65, p-STAT1 and p-JNK, but has no significant effect on p-P38 activity (Fig. 5A). TNFα, IFNγ and Anisomycin, specific agonists of NFκB, JAK/STAT and JNK/MAPK pathways, respectively, were utilized to determine the specific effects of HEV ORF3. Activation of p-P65, p-STAT1 and p-JNK was significantly impaired in ORF3-expressing cells, even following treatment with the respective agonists (Fig. 5B). Since these signaling pathways play crucial roles in regulation of TLR7, inhibition of NF-κB, JAK/STAT and JNK/MAPK pathways by ORF3 may contribute to the downregulation of TLR7 and generation of endogenous type I interferon.

## Discussion

HEV is the leading cause of acute viral hepatitis, which can potentially result in chronic hepatitis or even fulminant hepatic failure, especially in immunocompromised individuals or pregnant women^22^. This plus-stranded RNA virus exists in both (pseudo)-enveloped and non-enveloped forms and contains three ORFs^23,24^. Among these, HEV ORF3 encodes a phosphoprotein that plays critical roles in regulating a series of host cell processes during viral invasion and generating an immunosuppressive environment suitable for viral replication and pathogenesis. Since well-coordinated and tightly regulated signaling through innate and adaptive immune receptors is central to the development of an effective immune response, dysregulation of this process can lead to excessive inflammation and immunopathology^25,26^. Several viruses have evolved multiple mechanisms to acquire the ability to regulate the host immune system. However, limited studies to date have focused on the regulatory effects of HEV ORF3 on innate immunity.

HEV ORF3 not only inhibits the antiviral effect of IFNα by suppressing activation of STAT1 but also downregulates the TLR3-mediated signaling pathway, which may affect production of endogenous interferon^20,27^. IFNs, a multifunctional family of cytokines critical in the first line of defense against viral infection, are induced upon engagement of viral molecules. Viral nucleic acids are recognized by pattern recognition receptors, triggering host innate immune responses^28^. However, viruses have developed an extraordinary range of strategies to counteract host immune responses. In view of the current finding that generation of type I interferon is significantly inhibited in ORF3-expressing THP1 cells (Fig. 1A–C), we propose that ORF3 aids in HEV replication and evasion of host cell immune clearance through inhibiting the secretion of endogenous interferon, consistent with earlier studies.

The interferon system induced by TLRs is an important type of innate immune response^12^. To clarify the mechanisms underlying the ORF3-mediated decrease in IFN production, TLR3, TLR4 and TLR7 protein and mRNA levels in THP1 and LO2 cells overexpressing ORF3 proteins were examined. No significant differences in TLR4 expression were evident while TLR3 and TLR7 levels in ORF3-expressing cells were significantly lower relative to the control groups (Fig. 1C–E). Specific agonists of TLR3 and TLR7 were further used to promote activation of the corresponding signaling pathways and induce endogenous type I interferon production. Notably, ORF3 induced a significant decrease in the IFN content (Fig. 2A–D), downregulation of TLR3 and TLR7, and inhibition of the corresponding downstream factors including p-IRF3 and p-IRF7 (Fig. 2E,F).

The ORF3 protein is reported to exert a considerable inhibitory effect on TLR3-mediated NFκB signaling^20^, which may further influence the innate immune system. While the downstream signaling pathway of TLR7 has been relatively well characterized, its upstream regulatory mechanism is currently clear. Therefore, in the current study, cells were treated with both LPS and specific inhibitors of the appropriate signaling pathways to determine the regulatory mechanisms of TLR7 expression. Our results indicate that NFκB, JAK/STAT and JNK/MAPK pathways are critical for expression of TLR7 while ERK/MAPK and P38/MAPK do not participate this process (Fig. 3C,D). The NFκB family of proteins are key regulators of immune development, immune responses, inflammation, and cancer^29^. This network comprises five members (p65/RelA, RelB, cRel, p50, and p52), which form homodimers or heterodimers that differentially bind DNA. The dynamics of these proteins offer promising therapeutic targets that remain to be fully explored for ultimate translation into clinical practice^30^. Activation of transcription factors downstream of the NFκB pathway can promote the translational synthesis of numerous proteins involved in cellular communication and signal response paradigms^31^. Almost all mammalian and avian host cells have the ability to produce and respond to IFNs through engagement of cellular receptors that trigger the IFN effector response via the JAK/STAT signaling pathway. Induction of JAK/STAT signaling by IFNs leads to upregulation of multiple interferon-stimulated genes (ISGs) that rapidly kill viruses within infected cells. Viruses have evolved a range of strategies to counteract host immune responses via targeting JAK/STAT signaling^32^. Modulation of the JAK/STAT pathway by viruses through mechanisms involving ubiquitination, degradation and dephosphorylation is critical for establishment of chronic or persistent infections. The c-Jun N-terminal kinases (JNKs), members of the mitogen-activated protein kinase (MAPK) family, mediate eukaryotic cell responses to a range of abiotic and biotic stress insults^33^. The JNK pathway is activated in response to various stimuli, such as infection, inflammation, oxidative stress, DNA damage, osmotic stress or cytoskeletal changes, and plays evolutionarily conserved roles in numerous immune responses^34^. Earlier analyses of JNK-regulated pathways have revealed crucial roles in both cell proliferation and death^35,36^. Since JNK is a key component of innate immunity, bacteria, viruses and eukaryotic parasites can directly “tamper with” this pathway to evade or suppress immune responses^37–39^. In this study, we additionally predicted the binding sites and transcription factors of the tlr7 promoter region using PROMO software. Consequently, AP1, c-Jun, STAT1beta, RelA and NF-kappaB1 were identified as potential key activating transcription factors for the TLR7 promoter (Fig. 4). While some more accurate and credible technologies should be used to figure out the upstream regulatory mechanism of TLR7, such as dual-luciferase reporter system or oligopull down and EMSA.

The activation of NFκB, JAK/STAT and JNK/MAPK pathways in ORF3-expressing cells, either treated with agonists or left untreated, was significantly inhibited (Fig. 5A,B) while the P38/MAPK pathway was not affected. Furthermore, ERK/MAPK signaling was enriched in ORF3-expressing cells. Our results are consistent with those of earlier studies. For instance, ORF3 has been shown to inhibit NFκB signaling in the late phase of viral infection^9,20^. HEV inhibits IFNα signaling through binding of ORF3 protein to STAT1 in A549 cells^27^. ORF3 protein can also interact with Src Homology 3 (SH3) to activate the ERK/MAPK pathway that promotes survival of infected cells^40^. Accordingly, we hypothesize that HEV ORF3 regulates TLR7 expression through inducing downregulation of multiple signaling pathways, including NFκB, JAK/STAT and JNK/MAPK, thereby inhibiting endogenous IFN production.

In summary, HEV ORF3 protein inhibits endogenous type I IFN generation by suppressing expression of TLR3 and TLR7. Furthermore, the effects of ORF3 on TLR7 expression are mediated via impairment of multiple signaling pathways, including NFκB, JAK/STAT and JNK/MAPK. Data from our study provide novel insights into the networks connecting TLR signaling with NFκB, JAK/STAT as well as JNK/MAPK pathways and serve as a model for clarifying the mechanisms underlying pathogenesis and immune escape of HEV.

## Electronic supplementary material

Supplement S1.STR profiling report of THP-1 cell line.

Supplement S2.STR profiling report of HepG2 cell line.

Supplement S3.Initial pictures of western blots
