# Supplementary material for: HEV ORF3 downregulates TLR7 to inhibit the generation of type I interferon via impairment of multiple signaling pathways
Source: Sci Rep. 2018 Jun 5;8:8585. doi: 10.1038/s41598-018-26975-4 (PMC5988675; doi:10.1038/s41598-018-26975-4)
Supplement: Supplementary file 1 — Supplement S1.STR profiling report of THP-1 cell line. [file 41598_2018_26975_MOESM1_ESM.pdf]

HEV ORF3 downregulates TLR7 to inhibit the generation of type I interferon via impairment of multiple signaling pathways

Qingsong Lei[a], Lin Li[b], Shujun Zhang[a], Tianju Li[a], Xiaomei Zhang[a], Xiaolin Ding[a], Bo Qin[a, *]

武汉普诺赛生命科技有限公司

Procell Life Science&Technology Co,.Ltd.

附表 1：细胞株 THP-1 的 STR 位点和 Amelogenin 位点的基因分型结果

Schedule 1. Genotyping results of STR loci and Amelogenin loci of THP-1 cell line.

细胞 THP-1（图片编号为 PC74）		
Marker	Allele 1	Allele 2
D3S1358	15	17
**THO1**	8	9.3
D21S11	30	31.2
D18S51	13	14
Penta E	11	15
**D5S818**	11	12
**D13S317**	13	13
**D7S820**	10	10
**D16S539**	11	12
**CSF1PO**	11	13
Penta D	10	12
**AMEL**	X	Y
**vWA**	16	16
D8S1179	10	14
**TPOX**	8	11
FGA	24	25
D6S1043	14	14
D2S1338	17	18
D12S391	19	19
D19S433	12.2	13
D1S1656	16	17

附图 1：ATCC 官网 THP-1 细胞 STR 位点信息

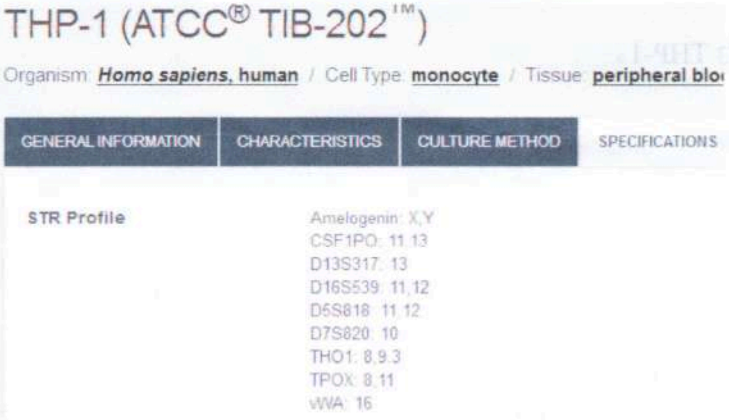
THP-1 (ATCC® TIB-202™)

Organism: Homo sapiens, human / Cell Type: monocyte / Tissue: peripheral blo

GENERAL INFORMATION | CHARACTERISTICS | CULTURE METHOD | SPECIFICATIONS

STR Profile

Amelogenin: X,Y
CSF1PO: 11,13
D13S317: 13
D16S539: 11,12
D5S818: 11,12
D7S820: 10
THO1: 8,9.3
TPOX: 8,11
vWA: 16

网站：www.procell.com.cn
电话：400-650-3656
邮箱：sales@procell.com.cn

附图 2：**THP-1 细胞 STR 位点和 Amelogenin 位点的基因分型结果**
Schedule 2. GeneMapper of STR loci and Amelogenin loci of THP-1 cell line.

PC74

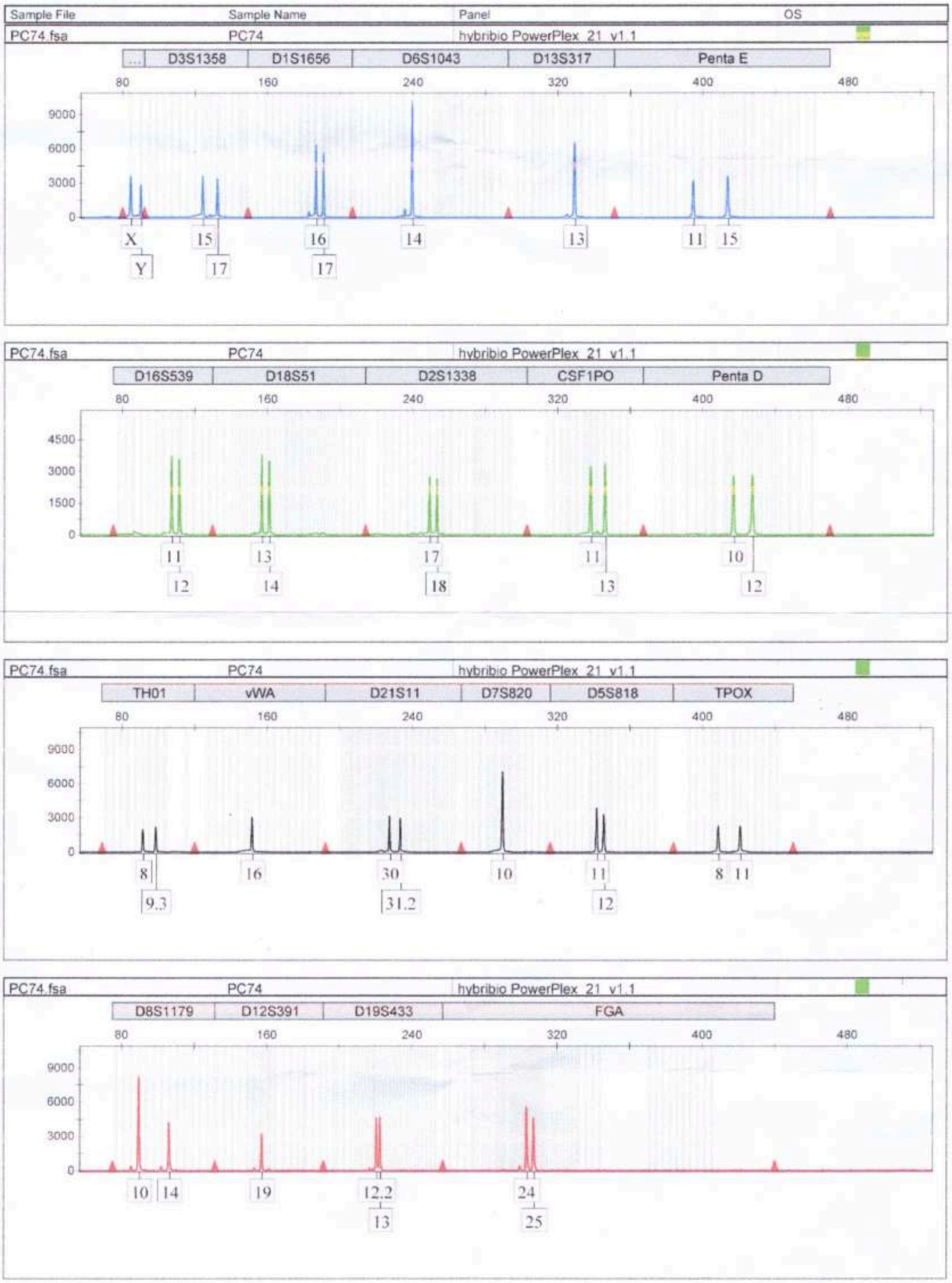

Sat May 13,2017 02:12PM, CST   Printed by: gm   Page 1

网站：www.procell.com.cn
电话：400-650-3656
邮箱：sales@procell.com.cn
