# Supplementary material for: HEV ORF3 downregulates TLR7 to inhibit the generation of type I interferon via impairment of multiple signaling pathways
Source: Sci Rep. 2018 Jun 5;8:8585. doi: 10.1038/s41598-018-26975-4 (PMC5988675; doi:10.1038/s41598-018-26975-4)
Supplement: Supplementary file 2 — Supplement S2.STR profiling report of HepG2 cell line. [file 41598_2018_26975_MOESM2_ESM.pdf]

HEV ORF3 downregulates TLR7 to inhibit the generation of type I interferon via impairment of multiple signaling pathways

Qingsong Lei[a], Lin Li[b], Shujun Zhang[a], Tianju Li[a], Xiaomei Zhang[a], Xiaolin Ding[a], Bo Qin[a, *]

Report of Human Cell Line Authentication

Delivery Date: 11-2, 2016

Analysis Date: 11-7, 2016

Ⅰ. Sample

Sample was labeled as HepG2.

Ⅱ. Method and Procedure

1. DNA was extracted by Chelex method.
2. PCR is amplified with STR Multi-amplification Kit (Goldeneye^{TM}20A ID System);
3. PCR products are assayed with ABI 3730xl DNA Analyzer (Applied Biosystems, Thermo Fisher Scientific, USA).
4. Data were analyzed using GeneMapper3.2 software and then compared with the ATCC and DSMZ databases for reference matching.

□. Results

The STR profiles of the cell line sample and the matching results are in the excel table.

HepG2:

1) No cross-contamination of other human cell line is found.
2) The submitted profile is an 100% match for the following ATCC human cell line(s) in the ATCC STR database (8 core loci plus Amelogenin): **HC-04**. 100% matched cell lines are found in DSMZ data bank. And the cell line named as **GS-HepG2**.

Notes：

1. Based on the ANSI Standard, cell lines with ≥80% match are considered to be related; i.e. derived from a common ancestry. Cell lines with between a 55% to 80% match require futher profiling for authentication of relatedness.

2. This data and analysis are for research use only.

Table: STR profiles of xxxx cell line

Marker	Allele 1	Allele 2
D19S433	15.2	
D5S818	11	12
D21S11	29	31
D18S51	13	14
D6S1043	13	
D3S1358	15	16
D13S317	9	13
D7S820	10	
D16S539	12	
CSF1PO	10	11
Penta D	9	13
AMEL	X	Y
vWA	17	
D8S1179	15	16
TPOX	8	9
Penta E	15	20
TH01	9	
D12S391	21	25
D2S1338	19	20
FGA	22	25

Figure: STR profiles of HepG2 cell line

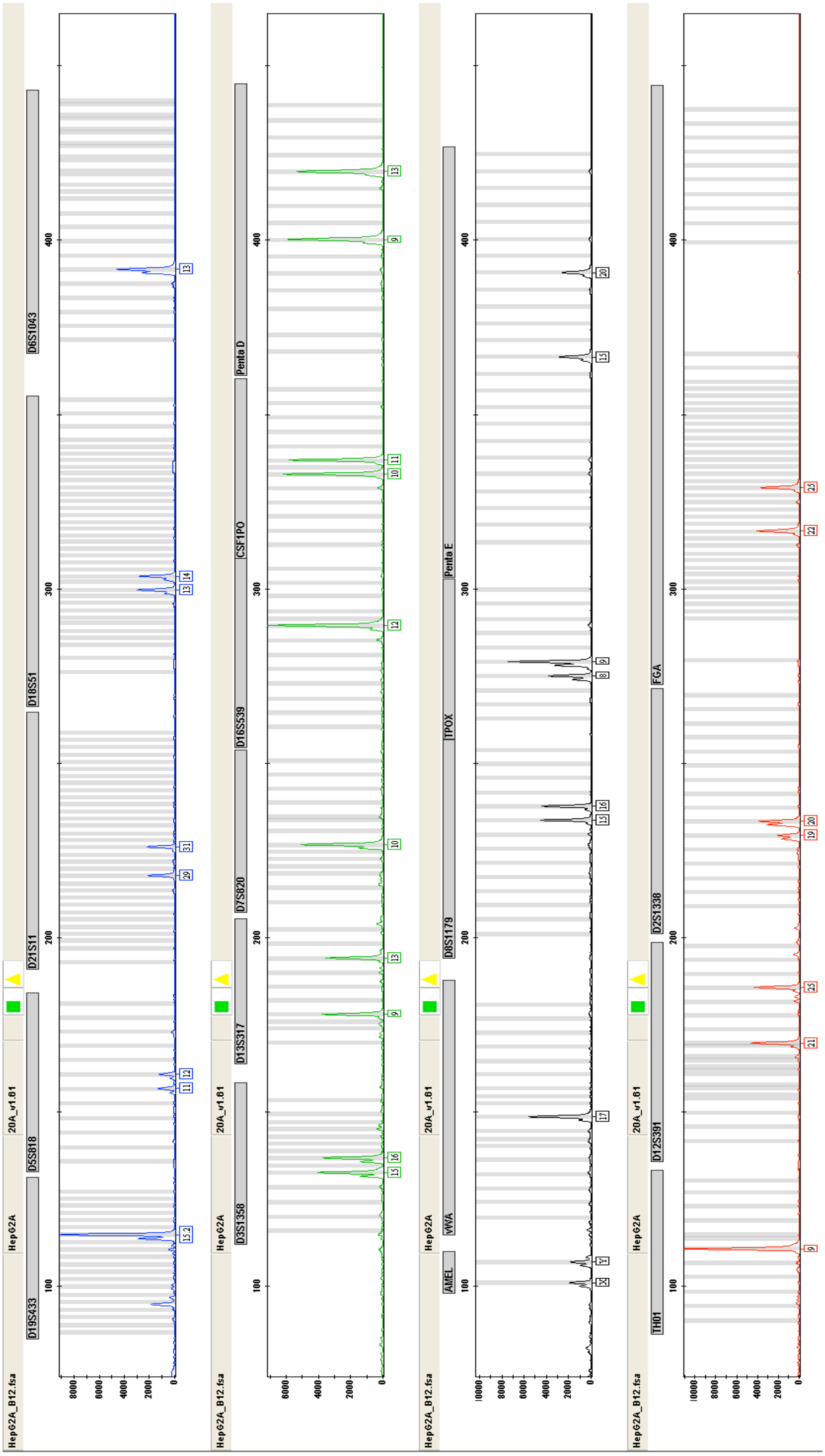

Figure: 100% matched cell lines are found in DSMZ data bank.

EV	Cell No.	Cell name	Locus names								
			D5S818	D13S317	D7S820	D16S539	VWA	TH01	AM	TPOX	CSF1PO
		Query (Your Cell)	*11,12*	*9,13*	*10,10*	*12,12*	*17,17*	*9,9*	*x,y*	*8,9*	*10,11*
1.00(36/36)	RCB1681	GS-HepG2	11,12	9,13	10,10	12,12	17,17	9,9	X,Y	8,9	10,11
0.94(34/36)	180	HEP-G2	11,12	9,13	10,10	12,13	17,17	9,9	X,Y	8,9	10,11
0.94(34/36)	CRL-11997	HEP G2/2.2.1	11,12	9,13	10,10	12,13	17,17	9,9	X,Y	8,9	10,11
0.94(34/36)	HB-8065	Hep G2	11,12	9,13	10,10	12,13	17,17	9,9	X,Y	8,9	10,11
0.94(34/36)	HB-8065.1	HepG2/SF	11,12	9,13	10,10	12,13	17,17	9,9	X,Y	8,9	10,11
0.94(34/36)	JCRB1054	Hep G2	11,12	9,13	10,10	12,13	17,17	9,9	X,Y	8,9	10,11
0.94(34/36)	RCB1648	Hep G2	11,12	9,13	10,10	12,13	17,17	9,9	X,Y	8,9	10,11
0.94(34/36)	RCB1886	Hep G2	11,12	9,13	10,10	12,13	17,17	9,9	X,Y	8,9	10,11
0.89(32/36)	CRL-10741	C3A [HepG2/C3A, derivative of Hep G2 (ATCC HB-8065)]	11,13	9,13	10,10	12,13	17,17	9,9	X,Y	8,9	10,11
