# Supplementary material for: HEV ORF3 downregulates TLR7 to inhibit the generation of type I interferon via impairment of multiple signaling pathways
Source: Sci Rep. 2018 Jun 5;8:8585. doi: 10.1038/s41598-018-26975-4 (PMC5988675; doi:10.1038/s41598-018-26975-4)
Supplement: Supplementary file 3 — Supplement S3.Initial pictures of western blots [file 41598_2018_26975_MOESM3_ESM.pdf]

HEV ORF3 downregulates TLR7 to inhibit the generation of type I interferon via impairment of multiple signaling pathways

Qingsong Lei[a], Lin Li[b], Shujun Zhang[a], Tianju Li[a], Xiaomei Zhang[a], Xiaolin Ding[a], Bo Qin[a, *]

Initial pictures of western blot in this article. These pictures were merged as below.

Chemi Hi Resolution	+	Colorimetric	=	Merge
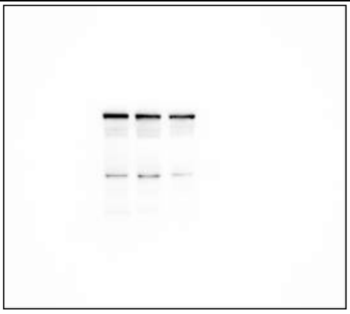	+	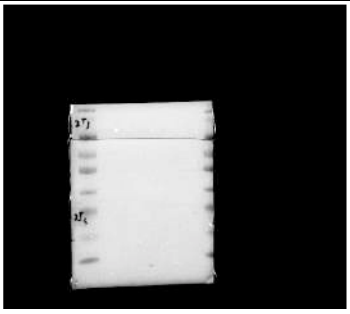	=	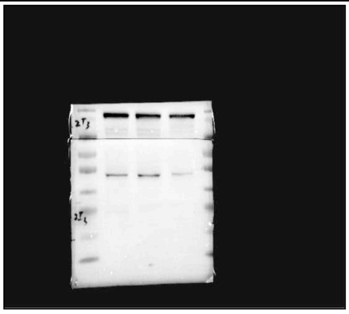

Fig 1

TLR3	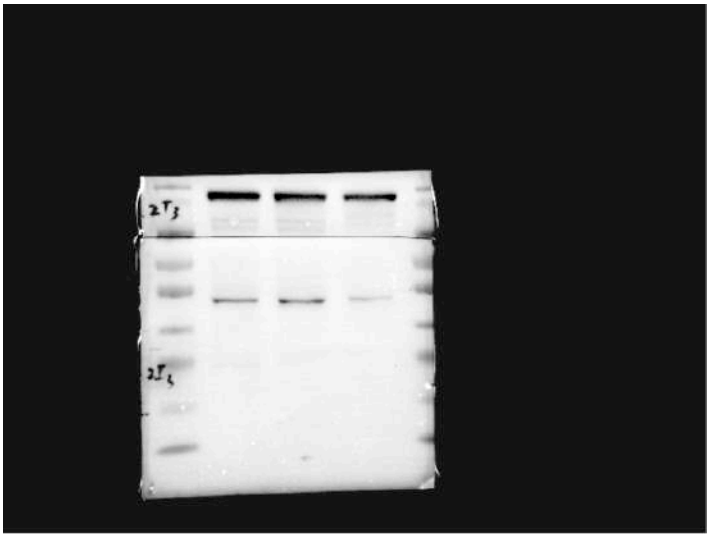
TLR4+ GAPDH	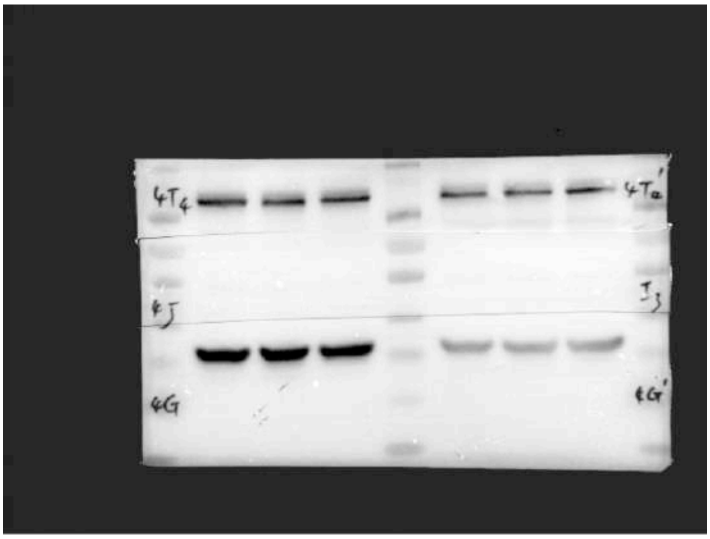

TLR7	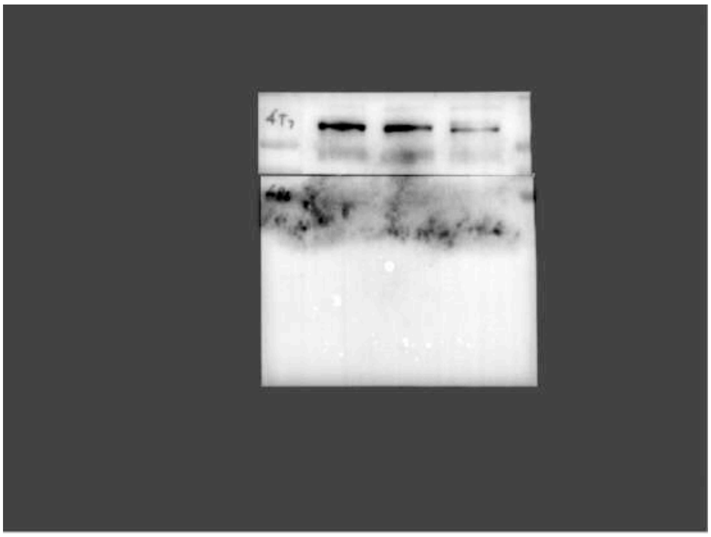
GFP+ pORF3	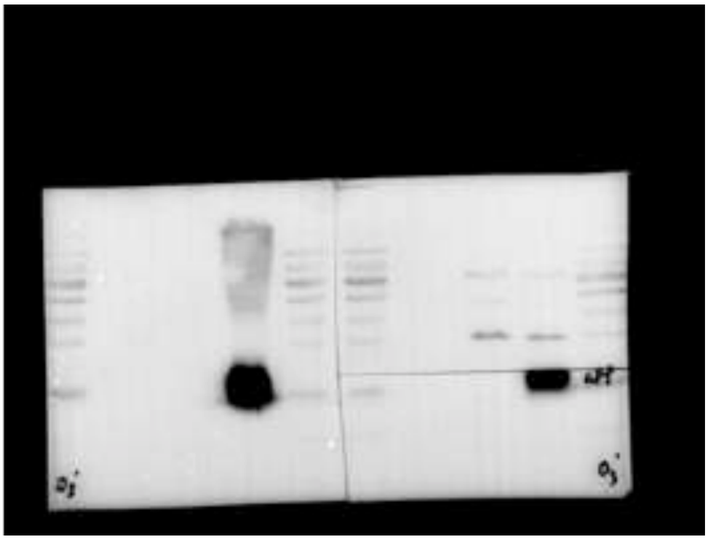

Fig 2

TLR3+<br>p-IRF3	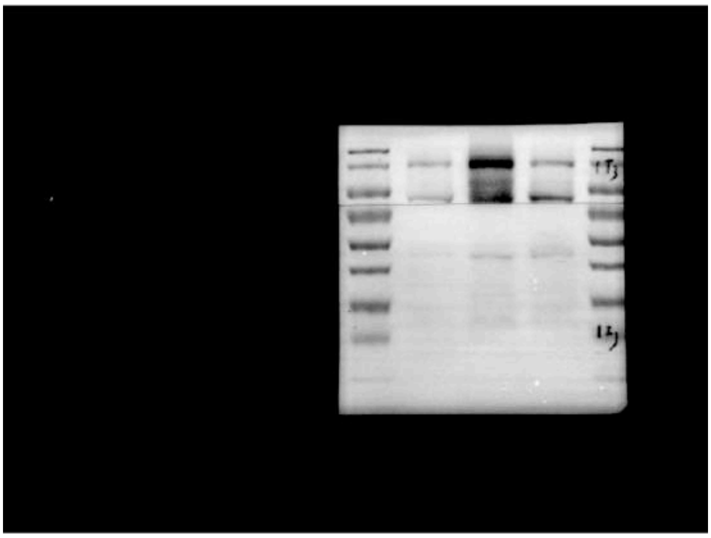
IRF3	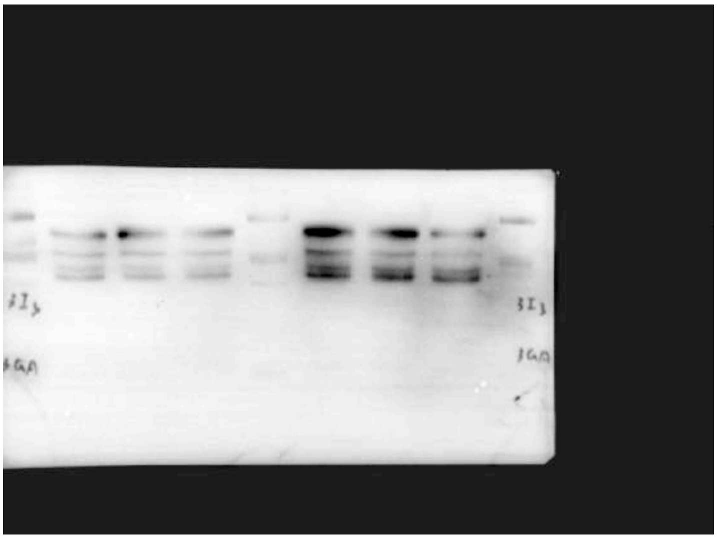
TLR7	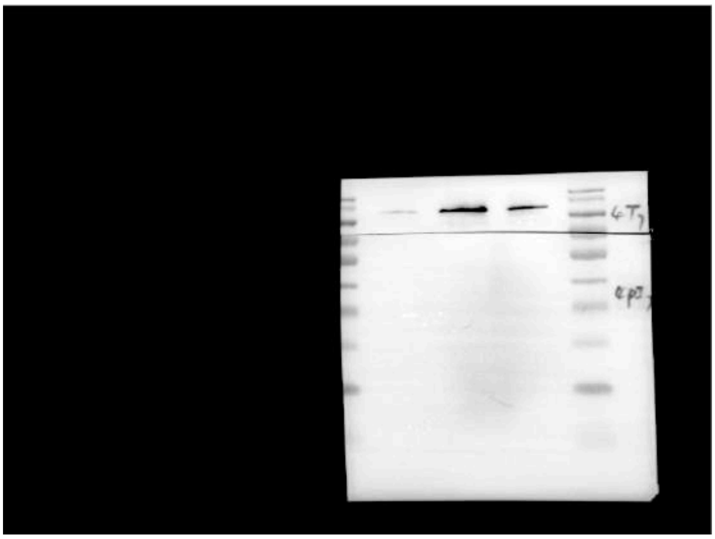

p-IRF7	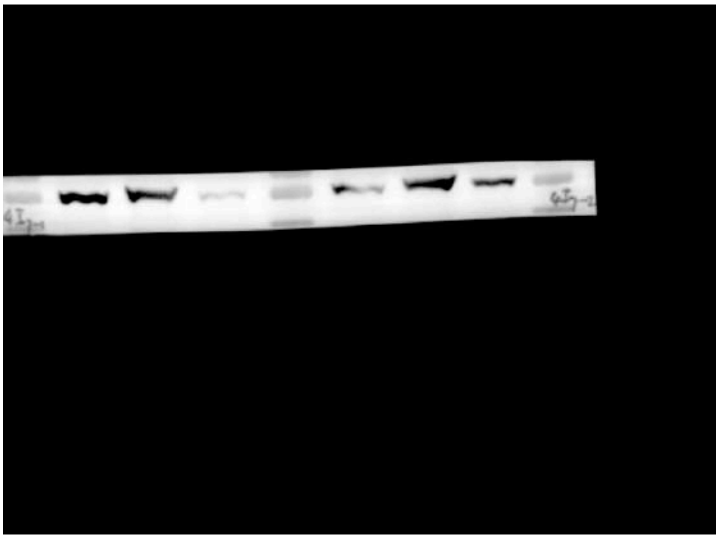
IRF7	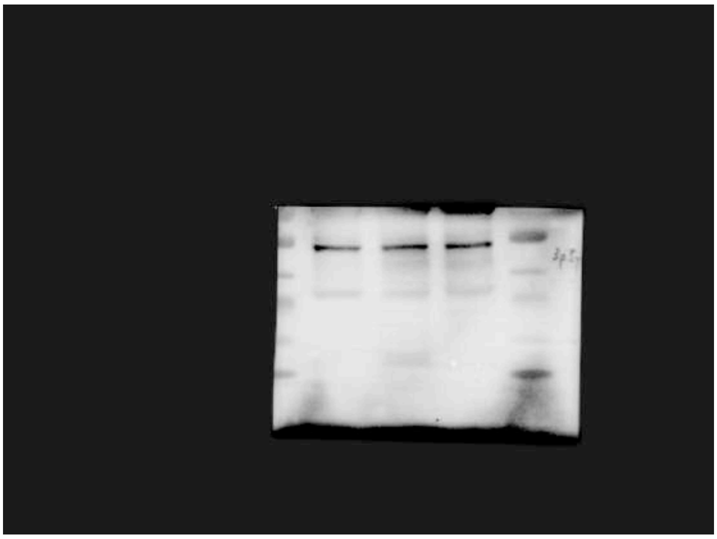
GAPDH	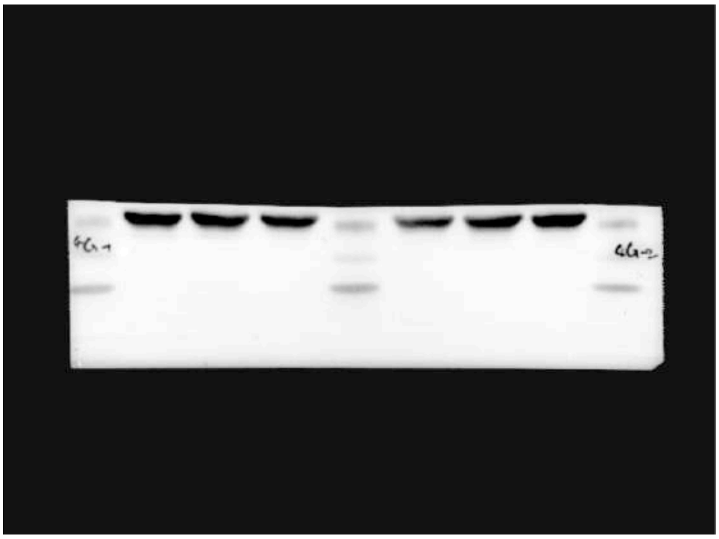

Fig 3

TLR7	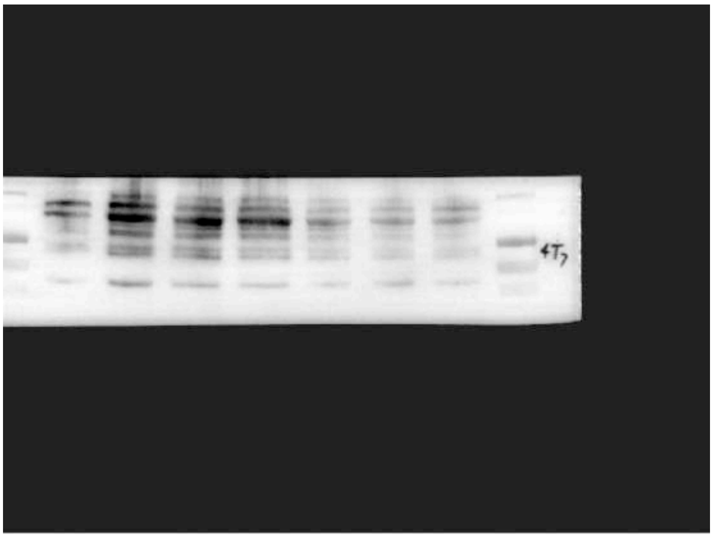
p-STA<br>T1	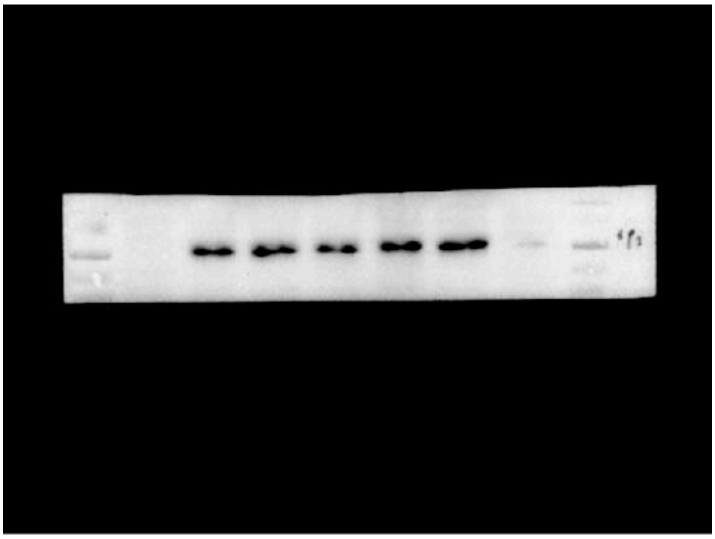
STAT1	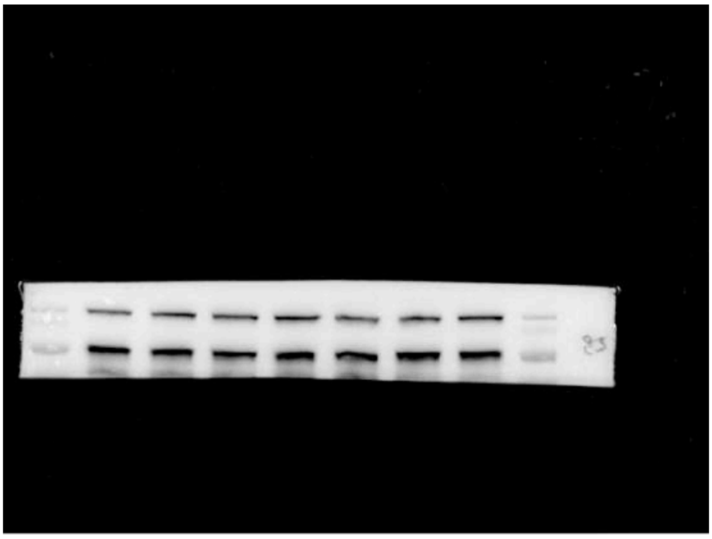

p-P65	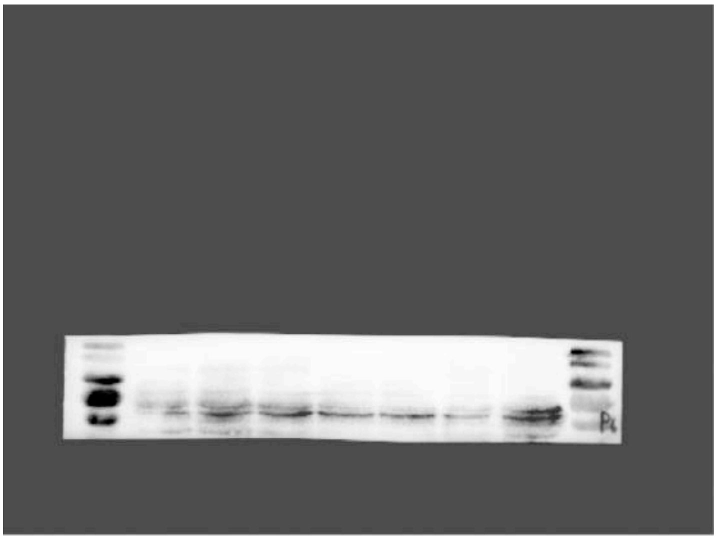
P65	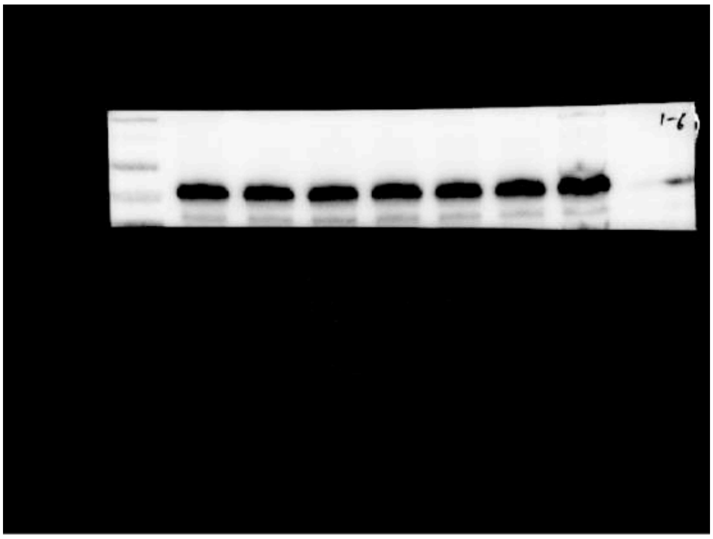
p-JNK	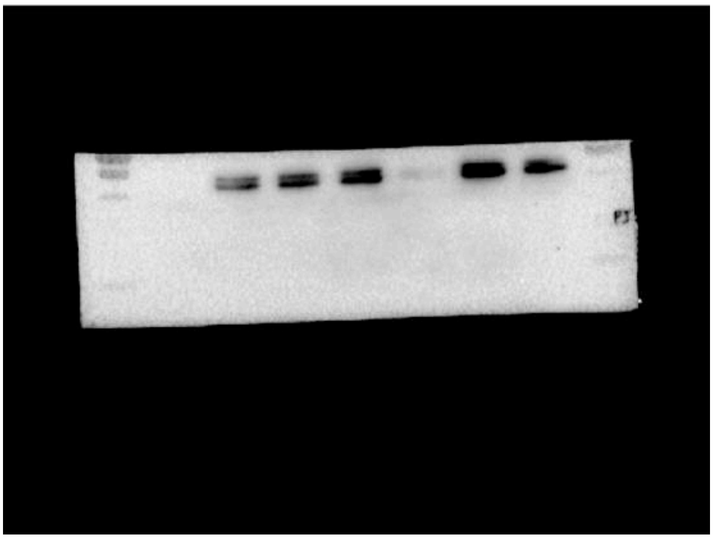

JNK	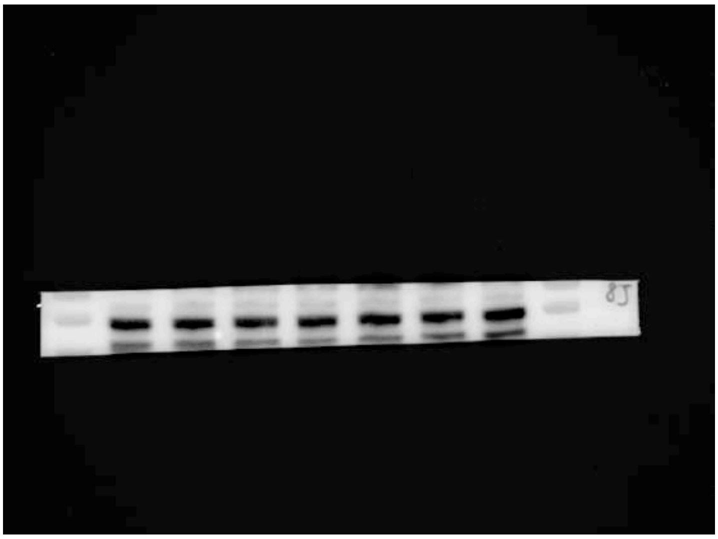
p-P38	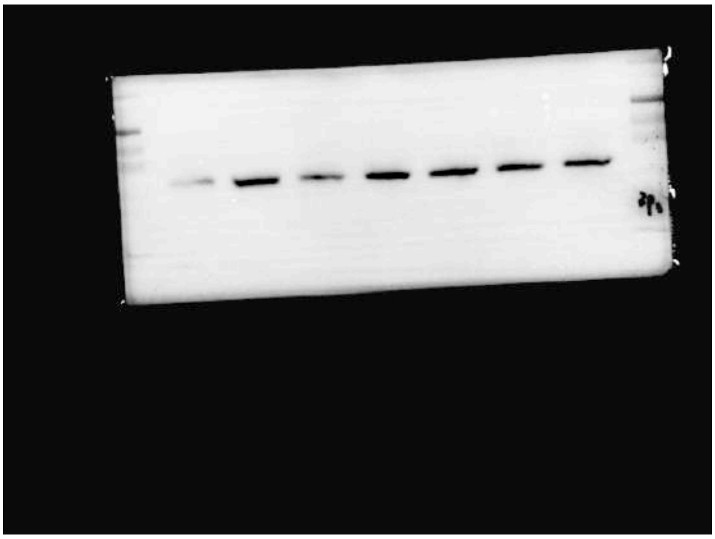
P38	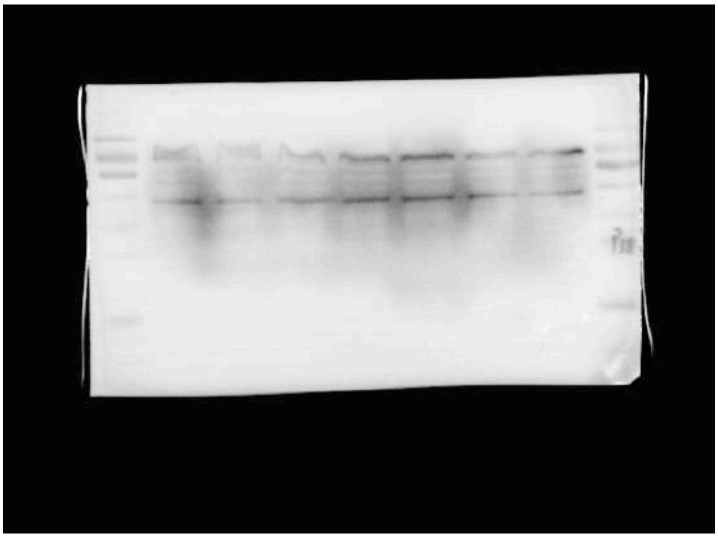

p-ERK	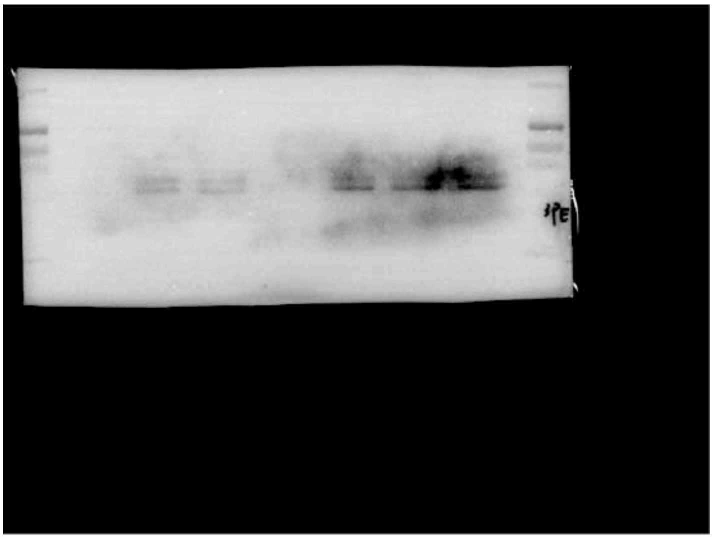
ERK	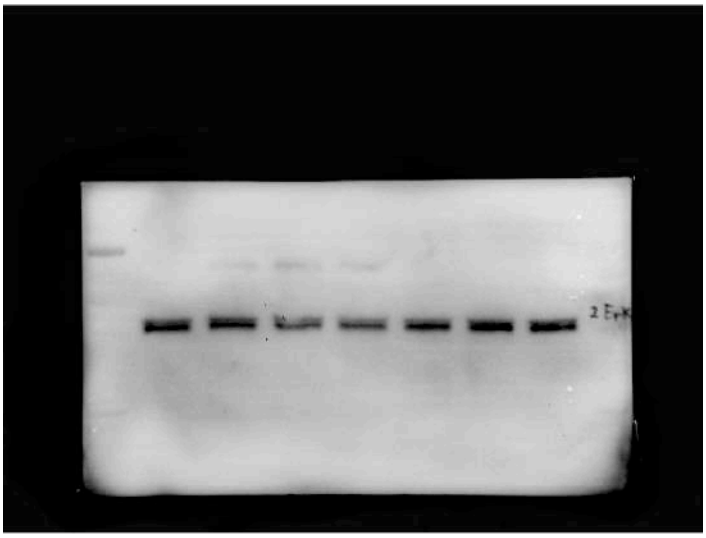
GAPDH	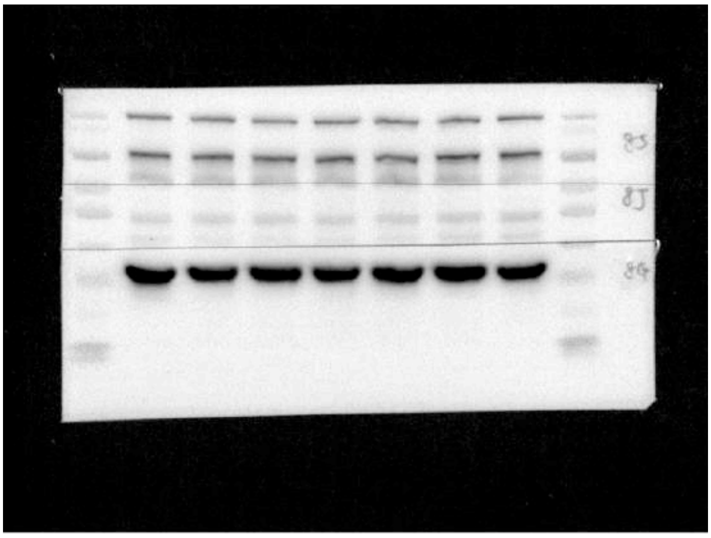

Fig 5

p-STAT1	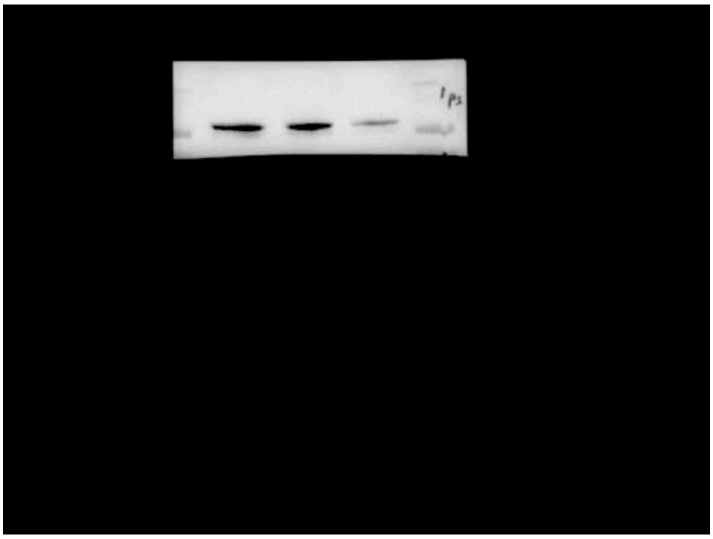
STAT1	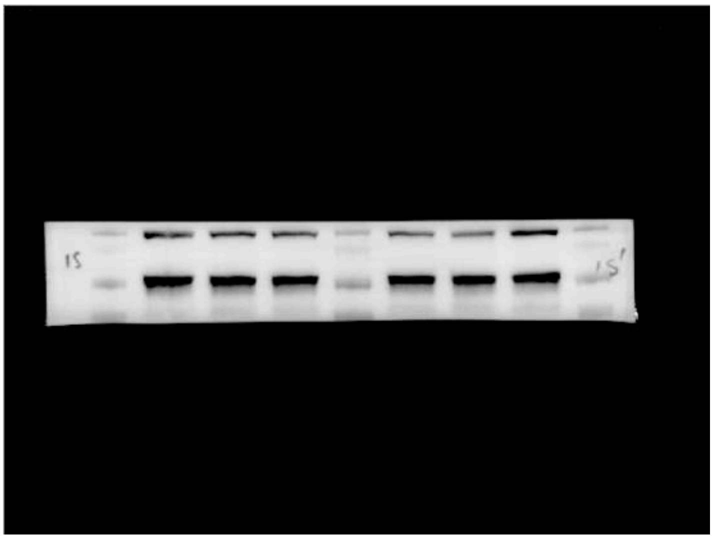
p-P65	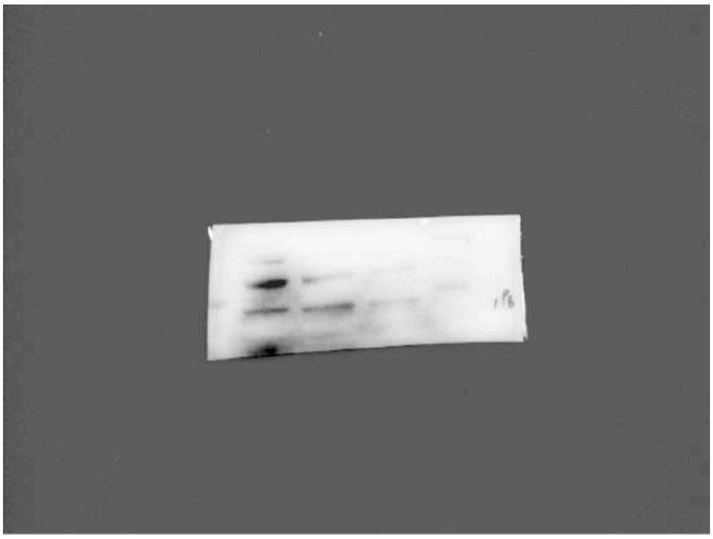

P65	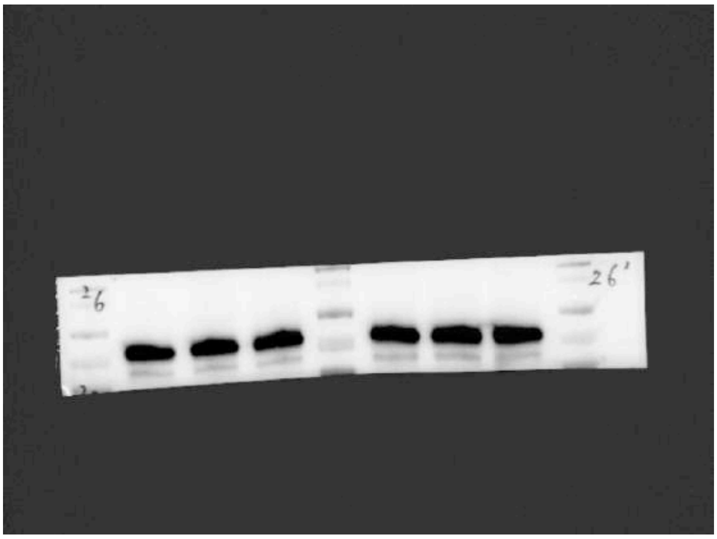
p-ERK	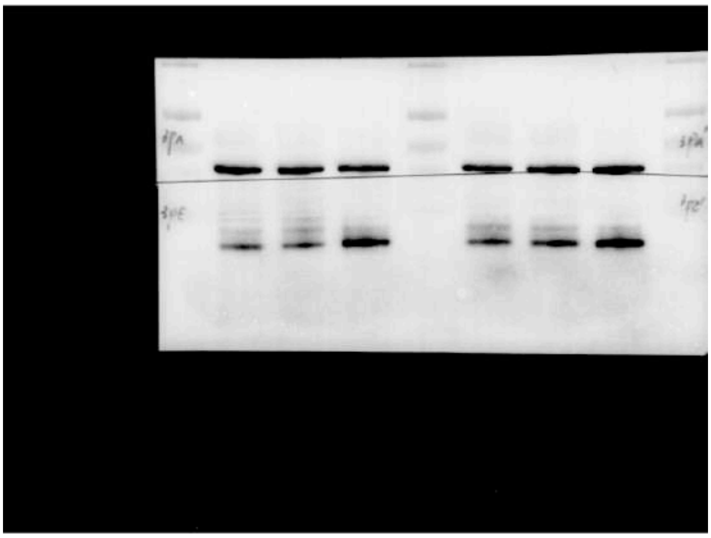
ERK	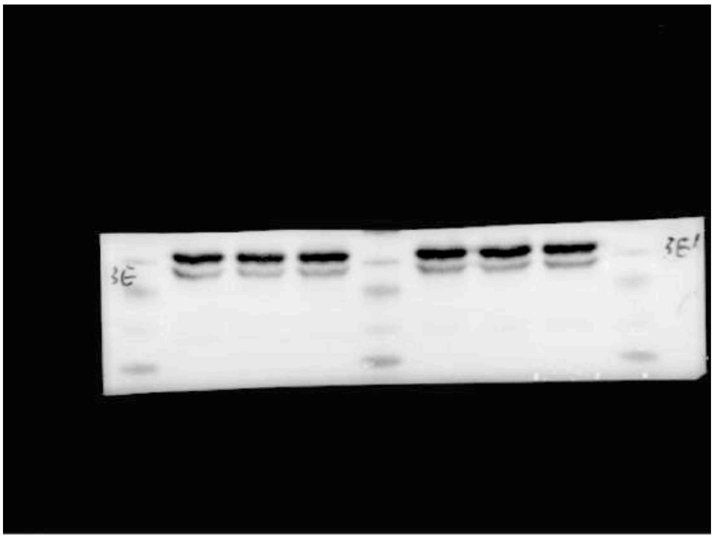

p-JNK	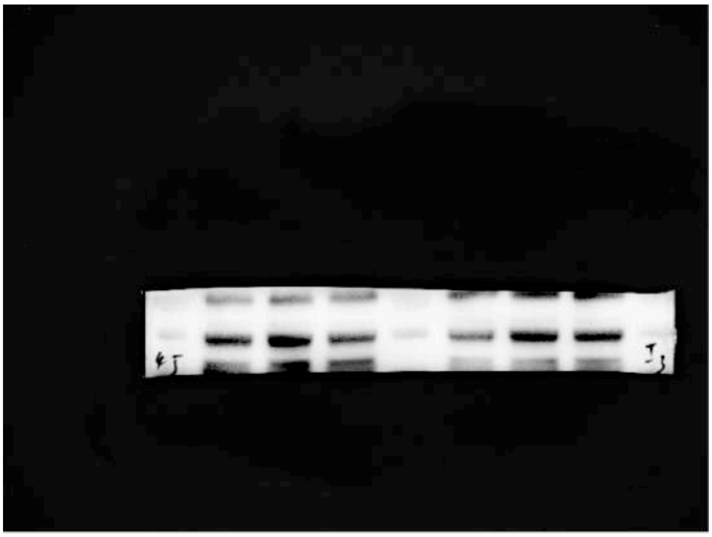
JNK	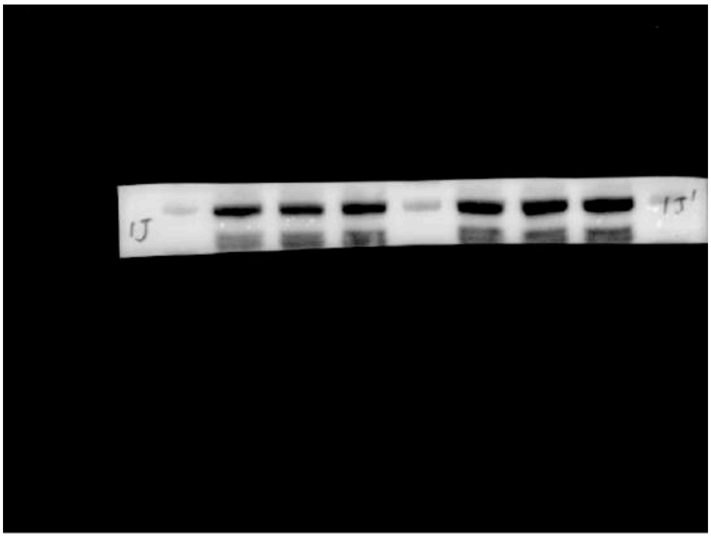
p-P38	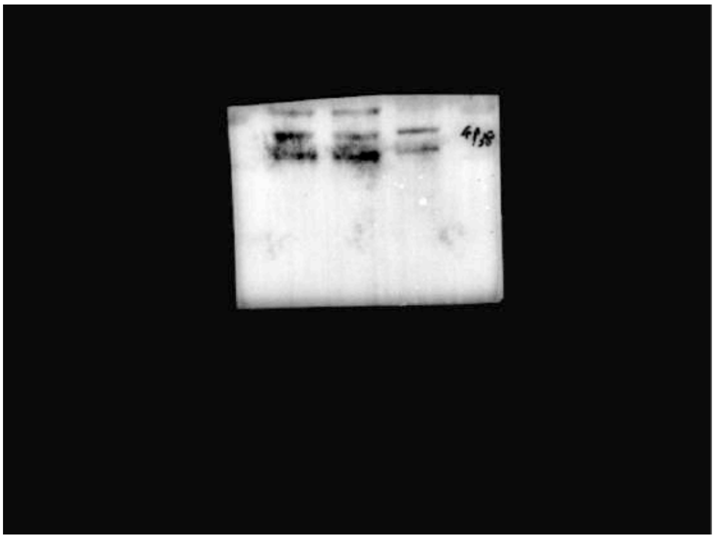

P38	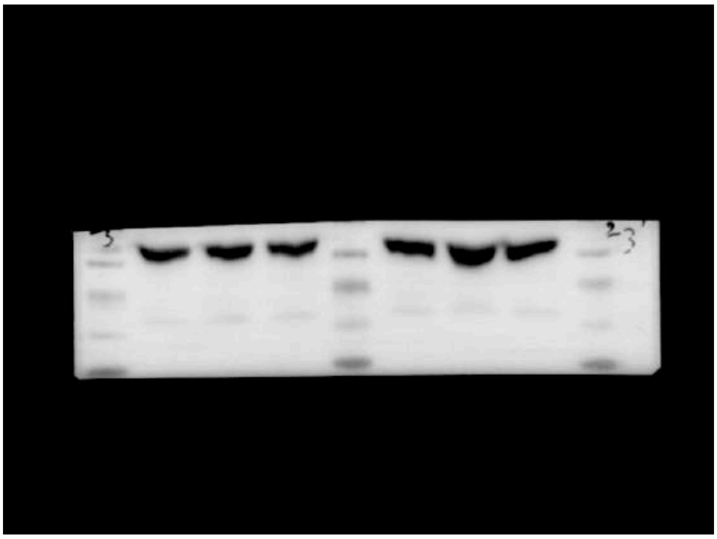
GAPDH	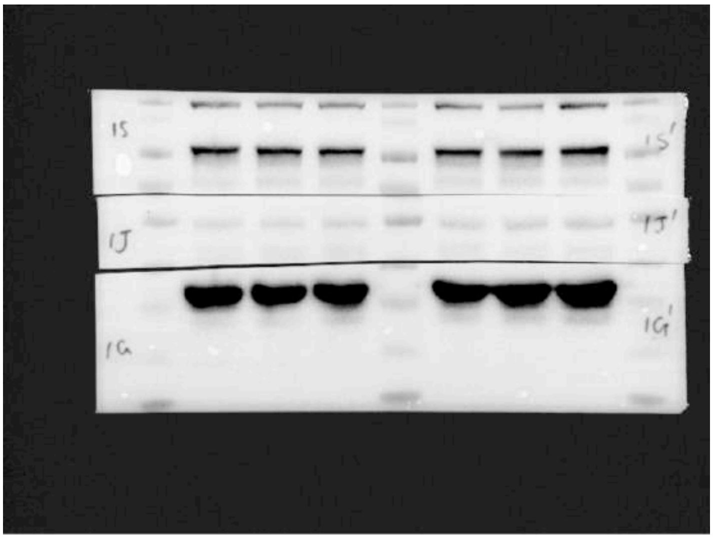
p-STAT1	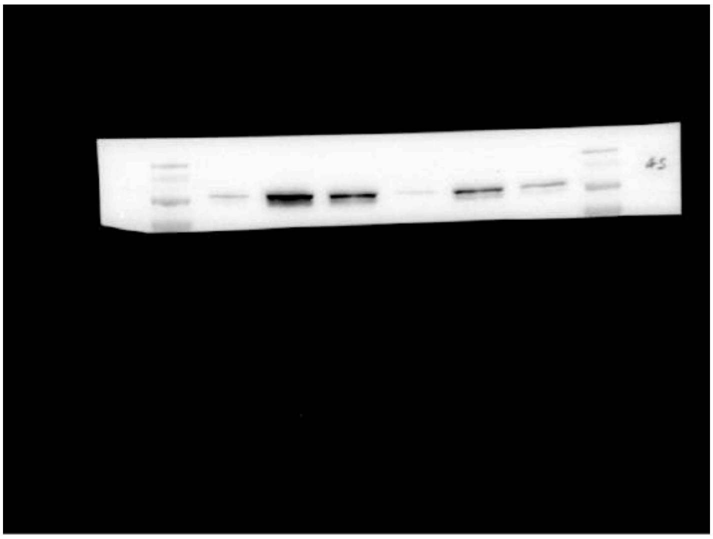

STAT1+ GAPDH	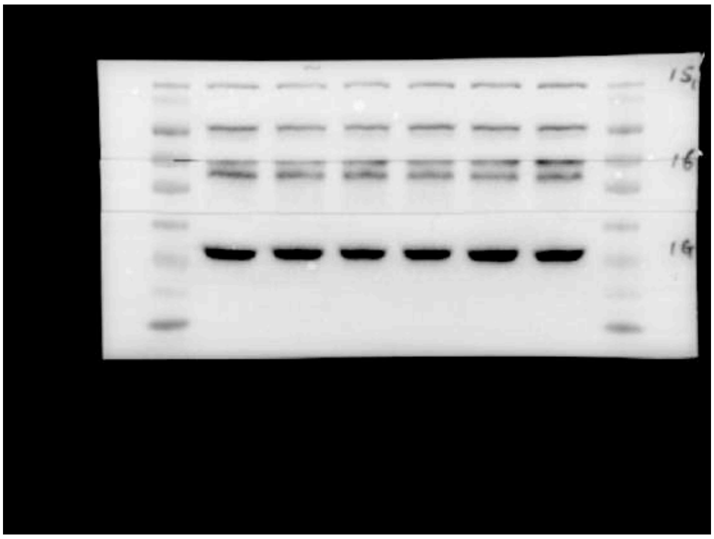
p-P65	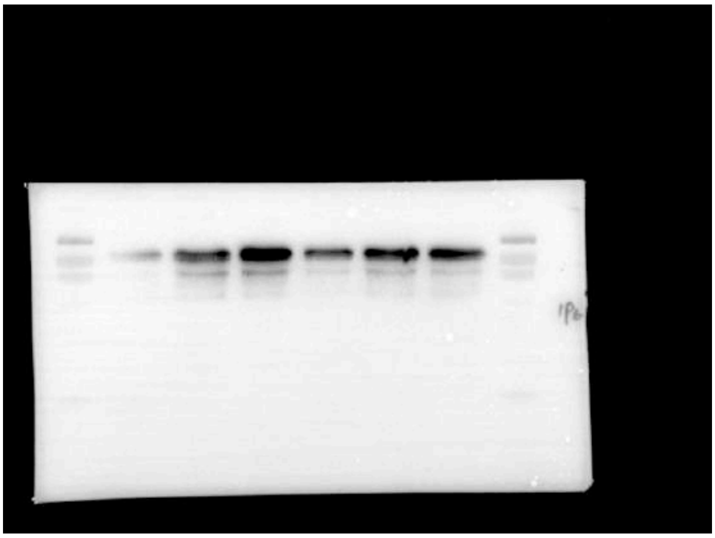
P65	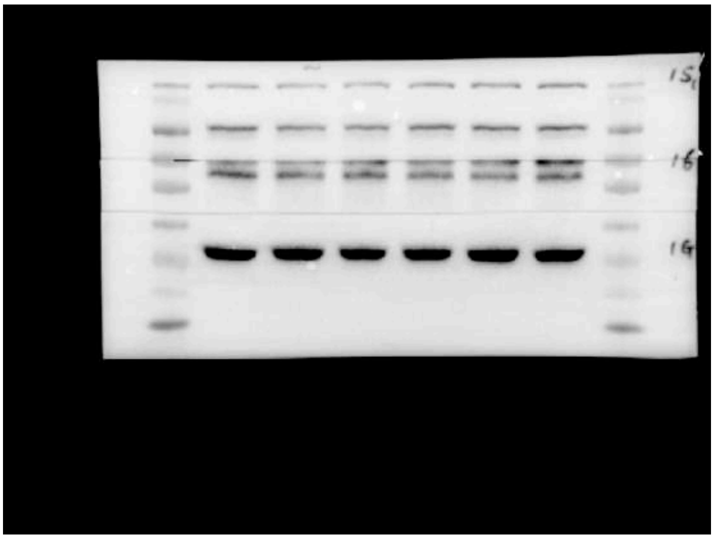

GAPDH	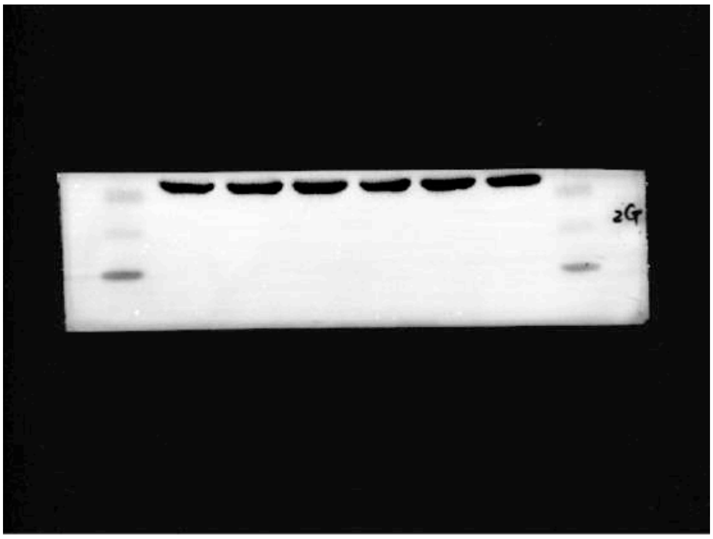
p-JNK	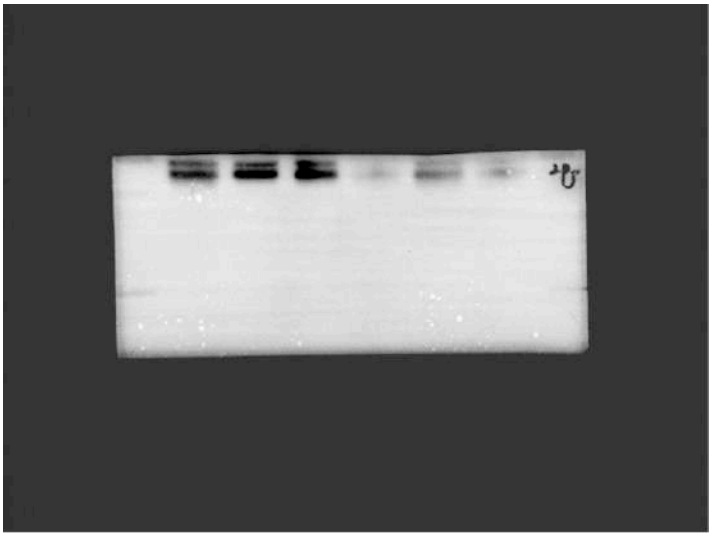
JNK	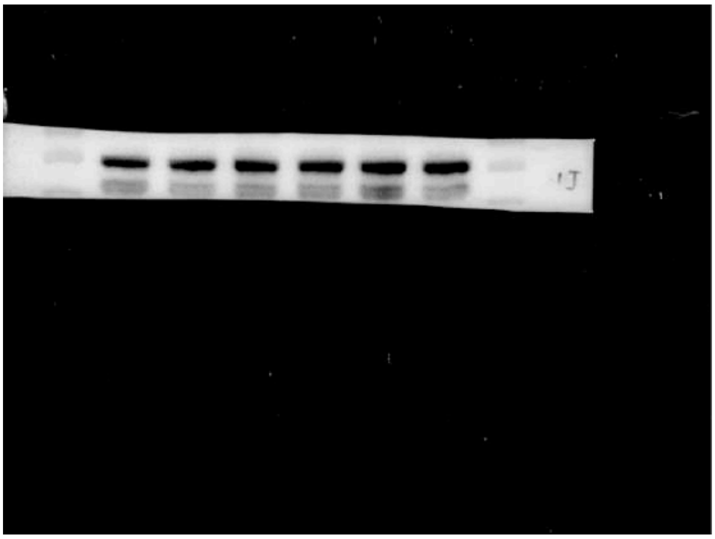

GAPDH	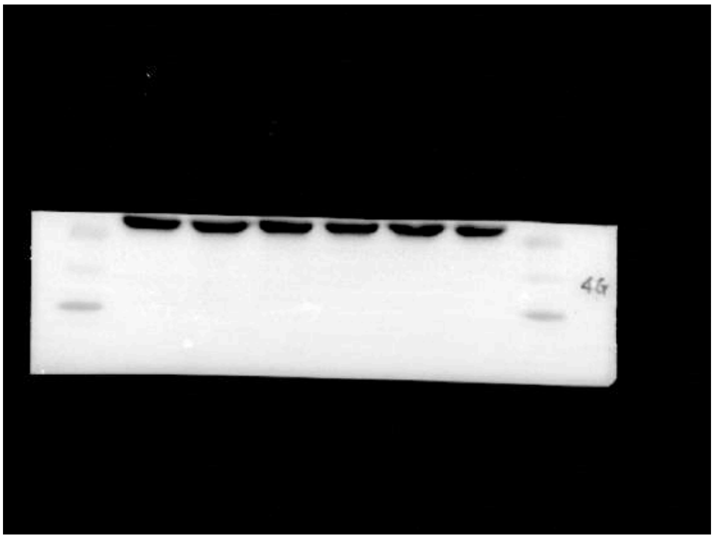
